# Supplementary material for: Digging into the NGS Information from a Large-Scale South European Population with Metastatic/Unresectable Pancreatic Ductal Adenocarcinoma: A Real-World Genomic Depiction
Source: Cancers (Basel). 2023 Dec 19;16(1):2. doi: 10.3390/cancers16010002 (PMC10778112; doi:10.3390/cancers16010002)
Supplement: Supplementary file 1 [file cancers-16-00002-s001.zip › Supplementary Tables.pdf]

Supplementary Table S1. 513 panel gene list

**206 full exon coverage and CNV Loss genes**									
ABRAXAS1	ACVR1B	ACVR2A	ADAMTS12	ADAMTS2	AMER1	APC	ARHGAP35	ARID1A	ARID1B
ARID2	ARID5B	ASXL1	ASXL2	**ATM**	**ATR**	**ATRX**	AXIN1	AXIN2	B2M
**BAP1**	**BARD1**	BCOR	**BLM**	BMPR2	**BRCA1**	**BRCA2**	**BRIP1**	CASP8	CBFB
CD274	CD276	CDC73	CDH1	CDH10	**CDK12**	CDKN1A	CDKN1B	CDKN2A	CDKN2B
CDKN2C	**CHEK1**	**CHEK2**	CIC	CREBBP	CSMD3	CTCF	CTLA4	CUL3	CUL4A
CUL4B	CYLD	CYP2C9	DAXX	DDX3X	DICER1	DNMT3A	DOCK3	DPYD	DSC1
DSC3	ELF3	ENO1	EP300	EPCAM	EPHA2	ERAP1	ERAP2	ERCC2	ERCC4
ERRFI1	ETV6	**FANCA**	**FANCC**	**FANCD2**	**FANCE**	**FANCF**	**FANCG**	**FANCI**	**FANCL**
**FANCM**	FAT1	FBXW7	FUBP1	GATA3	GNA13	GPS2	HDAC2	HDAC9	HLA-A
HLA-B	HNF1A	INPP4B	JAK1	JAK2	JAK3	KDM5C	KDM6A	KEAP1	KMT2A
KMT2B	KMT2C	KMT2D	LARP4B	LATS1	LATS2	MAP2K4	MAP2K7	MAP3K1	MAP3K4
MAPK8	MEN1	MGA	MLH1	MLH3	**MRE11**	MSH2	MSH3	MSH6	MTAP
MUTYH	**NBN**	NCOR1	NF1	NF2	NOTCH1	NOTCH2	NOTCH3	NOTCH4	**PALB2**
PARP1	PARP2	PARP3	PARP4	PBRM1	PDCD1	PDCD1LG2	PDIA3	PGD	PHF6
PIK3R1	PMS1	PMS2	POLD1	POLE	POT1	PPM1D	**PPP2R2A**	PRDM1	PRDM9
PRKAR1A	PTCH1	PTEN	PTPRT	**RAD50**	**RAD51**	**RAD51B**	**RAD51C**	**RAD51D**	**RAD52**
**RAD54L**	RASA1	RASA2	RB1	RBM10	RECQL4	RNASEH2A	RNASEH2B	RNF43	RPA1
RUNX1	SDHA	SDHB	SDHD	SETD2	SLX4	SMAD2	SMAD4	SMARCA4	SMARCB1
SOX9	SPEN	STAG2	STK11	SUFU	TAP1	TAP2	TBX3	TCF7L2	TET2
TGFBR2	TNFAIP3	TNFRSF14	TP53	TP63	TPP2	TSC1	TSC2	USP9X	VHL
WT1	**XRCC2**	**XRCC3**	ZFHX3	ZMYM3	ZRSR2				
**21 full exon coverage genes**									
CALR	CIITA	CYP2D6	ERCC5	FAS	ID3	KLHL13	MTUS2	PSMB10	PSMB8
PSMB9	RNASEH2C	RPL22	RPL5	RUNX1T1	SDHC	SOCS1	STAT1	TMEM132D	UGT1A1
ZBTB20									
**108 Hotspot and CNV genes**									
ABL1	ABL2	AKT1	AKT2	AKT3	ALK	AR	ARAF	AURKA	AURKC
AXL	BCL2	BCL2L12	BCL6	BRAF	CARD11	CBL	CCND1	CCND2	CCND3
CCNE1	CDK4	CDK6	CHD4	DDR2	EGFR	EIF1AX	ERBB2	ERBB3	ERBB4
ESR1	EZH2	FAM135B	FGFR1	FGFR2	FGFR3	FGFR4	FLT3	FLT4	FOXA1
GATA2	GNAS	H3F3A	H3F3B	IDH2	IKBKB	IL7R	KDR	KIT	KLF5
KRAS	MAGOH	MAP2K1	MAPK1	MAX	MDM4	MECOM	MEF2B	MET	MITF

MPL	MTOR	MYC	MYCN	MYD88	NFE2L2	NRAS	NTRK1	NTRK3	PCBP1
PDGFRA	PDGFRB	PIK3C2B	PIK3CA	PIK3CB	PIK3R2	PIM1	PLCG1	PPP2R1A	PPP6C
PRKACA	PTPN11	PXDNL	RAC1	RAF1	RARA	RET	RHEB	RICTOR	RIT1
ROS1	SETBP1	SF3B1	SLCO1B3	SMC1A	SMO	SPOP	SRC	STAT3	STAT6
TERT	TOP1	TPMT	U2AF1	USP8	XPO1	ZNF217	ZNF429		
**61 hotspot genes**									
ACVR1	ATP1A1	BCR	BMP5	BTK	CACNA 1D	CD79B	CSF1R	CTNNB1	CUL1
CYSLTR2	DGCR8	DROSHA	E2F1	EPAS1	FGF7	FOXL2	FOXO1	GLI1	GNA11
GNAQ	HIF1A	HIST1H2 BD	HIST1H3 B	HRAS	IDH1	IL6ST	IRF4	IRS4	KLF4
KNSTRN	MAP2K2	MED12	MYOD1	NSD2	NT5C2	NTRK2	NUP93	PAX5	PIK3CD
PIK3CG	PTPRD	RGS7	PIK3CD	PIK3CG	PTPRD	RGS7	RHOA	RPL10	SIX1
SIX2	SNCAIP	SOS1	SOX2	SRSF2	STAT5B	TAF1	TGFBR1	TRRAP	TSHR
WAS									
**19 CNV Gain genes**									
ABCB1	CTNND2	DDR1	EMSY	FGF19	FGF23	FGF3	FGF4	FGF9	FYN
GLI3	IGF1R	MCL1	MDM2	MYCL	RPS6KB 1	RPTOR	YAP1	YES1	
**86 TMB only genes**									
A1CF	ACSM2B	ADAM18	ANO4	ARMC4	BRINP3	C6	C8A	C8B	CANX
CASR	CD163	CNTN6	CNTNAP 4	CNTNAP5	COL11A 1	DCAF4L 2	DCDC1	GALNT17	GPR158
GRID2	HCN1	HLA-C	KCND2	KCNH7	KEL	KIR3DL1	KRTAP2- 1	KRTAP6- 2	LRRC7
MARCO	NLRC5	NOL4	NRXN1	NYAP2	OR10G 8	OR2G6	OR2L13	OR2L2	OR2L8
OR2M3	OR2T3	OR2T33	OR2T4	OR2W3	OR4A1 5	OR4C15	OR4C6	OR4M1	OR4M2
OR5D18	OR5F1	OR5L1	OR5L2	OR6F1	OR8H2	OR8I2	OR8U1	ORC4	PAK5
PCDH17	PDE1A	PDE1C	PLXDC2	POM121L 12	PPFIA2	RBP3	REG1A	REG1B	REG3A
REG3G	RPTN	RUNDC3 B	SH3RF2	SLC15A2	SLC8A1	SYT10	SYT16	TAPBP	TPTE
TRHDE	TRIM48	TRIM51	ZIM3	ZNF479	ZNF536				
**51 Fusion drivers genes**									
AKT2	ALK	AR	AXL	BRAF	BRCA1	BRCA2	CDKN2A	EGFR	ERBB2
ERBB4	ERG	ESR1	ETV1	ETV4	ETV5	FGFR1	FGFR2	FGFR3	FGR
FLT3	JAK2	KRAS	MDM4	MET	MYB	MYBL1	NF1	NOTCH1	NOTCH 4
NRG1	NTRK1	NTRK2	NTRK3	NUTM1	PDGFR A	PDGFRB	PIK3CA	PPARG	PRKAC A
PRKACB	PTEN	RAD51B	RAF1	RB1	RELA	RET	ROS1	RSPO2	RSPO3
TERT									

**Genes of the homologous recombination (HR) complex are in blue color

Supplementary Table S2. 513 panel gene list

87 hotspot genes									
AKT1	AKT2	AKT3	ALK	AR	ARAF	AXL	BRAF	BTK	CBL
CCND1	CDK4	CDK6	**CHEK2**	CSF1R	CTNNB1	DDR2	EGFR	ERBB2	ERBB3
ERBB4	ERCC2	ESR1	EZH2	FGFR1	FGFR2	FGFR3	FGFR4	FLT3	FOXL2
GATA2	GNA11	GNAQ	GNAS	H3F3A	HIST1H3B	HNF1A	HRAS	IDH1	IDH2
JAK1	JAK2	JAK3	KDR	KIT	KNSTRN	KRAS	MAGOH	MAP2K1	MAP2K2
MAP2K4	MAPK1	MAX	MDM4	MED12	MET	MTOR	MYC	MYCN	MYD88
NFE2L2	NRAS	NTRK1	NTRK2	NTRK3	PDGFRA	PDGFRB	PIK3CA	PIK3CB	PPP2R1A
PTPN11	RAC1	RAF1	RET	RHEB	RHOA	ROS1	SF3B1	SMAD4	SMO
SPOP	SRC	STAT3	TERT	TOP1	U2AF1	XPO1			
**48 full exon coverage**									
ARID1A	**ATM**	**ATR**	**ATRX**	**BAP1**	**BRCA1**	**BRCA2**	CDK12	CDKN1B	CDKN2A
CDKN2B	**CHEK1**	CREBBP	**FANCA**	**FANCD2**	**FANCI**	FBXW7	MLH1	**MRE11**	MSH2
MSH6	**NBN**	NF1	NF2	NOTCH1	NOTCH2	NOTCH3	**PALB2**	PIK3R1	PMS2
POLE	PTCH1	PTEN	**RAD50**	**RAD51**	**RAD51C**	**RAD51D**	**RAD51B**	RB1	RNF43
SETD2	SLX4	SMARCA4	SMARCB1	STK11	TP53	TSC1	TSC2		
**43 copy number variants**									
AKT1	AKT2	AKT3	ALK	AR	AXL	BRAF	CCND1	CCND2	CCND3
CCNE1	CDK2	CDK4	CDK6	EGFR	ERBB2	ESR1	FGF19	FGF3	FGFR1
FGFR2	FGFR3	FGFR4	FLT3	IGF1R	KIT	KRAS	MDM2	MDM4	MET
MYC	MYCL	MYCN	NTRK1	NTRK2	NTRK3	PDGFRA	PDGFRB	PIK3CA	PIK3CB
PPARG	RICTOR	TERT							
**51 fusion drivers**									
AKT2	ALK	AR	AXL	BRAF	**BRCA1**	**BRCA2**	CDKN2A	EGFR	ERBB2
ERBB4	ERG	ESR1	ETV1	ETV4	ETV5	FGFR1	FGFR2	FGFR3	FGR
FLT3	JAK2	KRAS	MDM4	MET	MYB	MYBL1	NF1	NOTCH1	NOTCH4
NRG1	NTRK1	NTRK2	NTRK3	NUTM1	PDGFRA	PDGFRB	PIK3CA	PPARG	PRKACA
PRKACB	PTEN	**RAD51B**	RAF1	RB1	RELA	RET	ROS1	RSPO2	RSPO3
TERT									

**Genes of the homologous recombination (HR) complex are in blue color

Supplementary Table S3. Study population and biomarkers'results obtained

Patient	Age	Gender	NGS results	PD-L1	TMB	MSI
1	39	Female	KRAS:c.35G>A (p.G12D)|			
2	68	Female				

3	57	Male	KRAS:c.35G>A (p.G12D)|ERBB2:c.1998_1999 delCT (p.L667fs*36)|CDKN2A:c.83_100delTGCGGGCGCTGCTGGAGG (-)|TP53:c.818G>A (p.R273H)|			
4	60	Female	TP53:c.380C>A (p.Ser127Tyr)|			
5	61	Male				
6	64	Male				
7	36	Female	KRAS:c.35G>A (p.G12D)|TP53:c.796G>A (p.G266R)|CCNE1:Amplification|			
8	61	Male	FGFR4:Amplification|			
9	60	Female	KRAS:c.34G>C (p.Gly12Arg)|TP53:c.817C>T (p.Arg273Cys)|			
10	71	Male	TP53:c.607G>A (p.Val203Met)|			
11	38	Male	TP53:c.557A>G (p.D186G)|			
12	48	Male	KRAS:c.35G>A (p.Gly12Asp)|TP53:c.215_216 insG (p.Val73fs)|			
13	63	Male	KRAS:c.35G>A (p.Gly12Asp)|TP53:c.430C>T (p.Gln144Ter)|			
14	64	Female	KRAS:c.35G>A (p.Gly12Asp)|			
15	54	Male	KRAS:c.35G>T (p.Gly12Val)|TP53:c.532C>T (p.His178Tyr)|ATM:c.9022C>T (p.Arg3008Cys)|TSC1:c.433C>T (p.Gln145Ter)|CREBBP:c.6019C>T (p.Gln2007Ter)|			

16	69	Male	KRAS:c.35G>T (p.Gly12Val)|			
17	48	Male	KRAS:c.35G>T (p.Gly12Val)|			
18	51	Male	TSC2:c.2380C>T (p.Q794*)|STK11:c.465-1G>A|STK11:c.465-1G>A|		32,24	
19	65	Female	KRAS:c.35G>A (p.Gly12Asp)|RNF43:c.2057_2058insG (p.Ser687fs)|Microsatellite Instability (MSI):STABLE (MSS)|PD-L1 expression:negative <1%|	negative| <1%	7,93	STABLE (MSS)
20	67	Male	KRAS:c.35G>T (p.Gly12Val)|		fail	
21	59	Female	KRAS:c.35G>A (p.Gly12Asp)|Microsatellite Instability (MSI):STABLE (MSS)|PD-L1 expression:negative insufficient material|	negative|insufficient material	12,2	STABLE (MSS)
22	47	Female	KRAS:c.35G>A (p.Gly12Asp)|TP53:c.706T>A (p.Tyr236Asn)|		11,81	
23	45	Female	KRAS:c.35G>T (p.Gly12Val)|		45,21	
24	81	Female	TP53:c.743G>A (p.Arg248Gln)|Microsatellite Instability (MSI):STABLE (MSS)|PD-L1 expression:negative <1%|	negative| <1%	13,17	STABLE (MSS)
25	63	Male	KRAS:c.35G>A (p.Gly12Asp)|		18,11	
26	62	Male	KRAS:c.35G>A (p.Gly12Asp)|TP53:c.823delT (p.Cys275fs)|		14,1	

<table>
<tr><td>27</td><td>73</td><td>Male</td><td>KRAS:c.34_35delGGinsCT (p.G12L)|CDK12:c.3283delG (p.E1095fs)|Microsatellite Instability (MSI):STABLE (MSS)|PD-L1 expression:fail fail|</td><td>fail|fail</td><td>21,67</td><td>STABLE (MSS)</td></tr>
<tr><td>28</td><td>55</td><td>Male</td><td>TP53:c.517G>A (p.Val173Met)|Microsatellite Instability (MSI):STABLE (MSS)|PD-L1 expression:fail fail|</td><td>fail|fail</td><td>18,08</td><td>STABLE (MSS)</td></tr>
<tr><td>29</td><td>67</td><td>Male</td><td>KRAS:c.37G>T (p.Gly13Cys)|</td><td></td><td>19,03</td><td></td></tr>
<tr><td>30</td><td>63</td><td>Male</td><td>KRAS:c.183A>C (p.Gln61His)|TP53:c.646G>A (p.Val216Met)|Microsatellite Instability (MSI):STABLE (MSS)|PD-L1 expression:negative <1%|</td><td>negative|<1%</td><td>18,97</td><td>STABLE (MSS)</td></tr>
<tr><td>31</td><td>71</td><td>Female</td><td>KRAS:c.35G>T (p.Gly12Val)|SMARCA4:c.2935C>T (p.Arg979Ter)|</td><td></td><td>13,1</td><td></td></tr>
<tr><td>32</td><td>78</td><td>Male</td><td>KRAS:c.35G>A (p.Gly12Asp)|PIK3R1:c.211G>T (p.Gly71Ter)|</td><td></td><td>22,27</td><td></td></tr>
<tr><td>33</td><td>53</td><td>Female</td><td>Microsatellite Instability (MSI):STABLE (MSS)|PD-L1 expression:negative insufficient material|</td><td>negative|insufficient material</td><td>20,99</td><td>STABLE (MSS)</td></tr>
<tr><td>34</td><td>58</td><td>Male</td><td>TP53:c.532C>T (p.His178Tyr)|</td><td></td><td>1,16</td><td></td></tr>
</table>

35	68	Male	KRAS:c.35G>A (p.Gly12Asp)|CDKN2A:c.221delA (p.Leu74fs)|TP53:c.659A>G (p.Tyr220Cys)|Microsatellite Instability (MSI):STABLE (MSS)|PD-L1 expression:positive 25%|	positive|25%	25,01	STABLE (MSS)
36	44	Female	KRAS:c.35G>T (p.Gly12Val)|CDKN2A:c.233_234delTC (p.Leu78fs)|RNF43:c.2057_2058insG (p.Ser687fs)|		32,1	
37	50	Male	KRAS:c.34G>C (p.Gly12Arg)|TP53:c.527G>T (p.Cys176Phe)|		23,76	
38	77	Female	KRAS:c.35G>T (p.Gly12Val)|POLE:c.6649C>T (p.Gln2217Ter)|		23,69	
39	68	Female	KRAS:c.35G>A (p.Gly12Asp)|TP53:c.646G>A (p.Val216Met)|		2,72	
40	28	Female	PIK3CA:c.3140A>G (p.H1047R)|MSH2:Loss (Oncogenic Mutations)|Microsatellite Instability (MSI):HIGH (MSI-High)|PD-L1 expression:positive 60%|	positive|60%	13,45	HIGH (MSI-High)
41	47	Male	TP53:c.211_220delCCCCCCGTGG (p.Pro72fs)|		2,8	
42	68	Male	Microsatellite Instability (MSI):STABLE (MSS)|PD-L1 expression:negative <1%|	negative|<1%	12,4	STABLE (MSS)
43	80	Female	KRAS:c.35G>T (p.Gly12Val)|		3,6	

<table>
<tr><td>44</td><td>68</td><td>Male</td><td>PALB2:c.1037_1041delAAGAA (p.Lys346Thrfs)|</td><td></td><td>8,03</td><td></td></tr>
<tr><td>45</td><td>48</td><td>Male</td><td>KRAS:c.35G>A (p.Gly12Asp)|Microsatellite Instability (MSI):STABLE (MSS)|PD-L1 expression:positive 2%|</td><td>positive|2 %</td><td>6,8</td><td>STABLE (MSS)</td></tr>
<tr><td>46</td><td>80</td><td>Male</td><td>KRAS:c.35G>A (p.Gly12Asp)|TP53:c.818G>A (p.Arg273His)|Microsatellite Instability (MSI):STABLE (MSS)|PD-L1 expression:negative <1%|</td><td>negative| <1%</td><td>0,94</td><td>STABLE (MSS)</td></tr>
<tr><td>47</td><td>75</td><td>Male</td><td>KRAS:c.35G>A (p.Gly12Asp)|</td><td></td><td>4,44</td><td></td></tr>
<tr><td>48</td><td>57</td><td>Male</td><td></td><td></td><td>1,68</td><td></td></tr>
<tr><td>49</td><td>54</td><td>Female</td><td>KRAS:c.34G>C (p.Gly12Arg)|</td><td></td><td>4,52</td><td></td></tr>
<tr><td>50</td><td>51</td><td>Female</td><td>KRAS:c.35G>T (p.Gly12Val)|Microsatellite Instability (MSI):STABLE (MSS)|PD-L1 expression:negative <1%|</td><td>negative| <1%</td><td>fail</td><td>STABLE (MSS)</td></tr>
<tr><td>51</td><td>44</td><td>Male</td><td>KRAS:c.34G>C (p.Gly12Arg)|TP53:c.916C>T (p.Arg306Ter)|Microsatellite Instability (MSI):STABLE (MSS)|PD-L1 expression:fail fail|</td><td>fail|fail</td><td>9,05</td><td>STABLE (MSS)</td></tr>
<tr><td>52</td><td>67</td><td>Male</td><td>KRAS:c.34G>C (p.Gly12Arg)|</td><td></td><td>5,18</td><td></td></tr>
</table>

53	43	Male	KRAS:c.38G>A (p.Gly13Asp)|TP53:c.524G>A (p.Arg175His)|Microsatellite Instability (MSI):STABLE (MSS)|PD-L1 expression:negative <1%|	negative|<1%	12,74	STABLE (MSS)
54	52	Male	KRAS:c.34G>C (p.Gly12Arg)|		6	
55	58	Female	FGFR1 fusion:WHSC1L1(1)-FGFR1(2)|Microsatellite Instability (MSI):STABLE (MSS)|PD-L1 expression:negative <1%|	negative|<1%	3,4	STABLE (MSS)
56	53	Female	KRAS:c.35G>T (p.Gly12Val)|SLX4:c.212C>G (p.Ser71Ter)|TP53:c.743G>A (p.Arg248Gln)|PPARG fusion:TSEN2(5)-PPARG(4)|		1,52	
57	61	Female	KRAS:c.35G>A (p.Gly12Asp)|		2,97	
58	59	Male	KRAS:c.35G>A (p.Gly12Asp)|TP53:c.725G>T (p.Cys242Phe)|RSPO2 fusion:EIF3E(1)-RSPO2(2)|Microsatellite Instability (MSI):STABLE (MSS)|PD-L1 expression:positive 30%|	positive|30%	5,44	STABLE (MSS)
59	66	Female	KRAS:c.35G>A (p.Gly12Asp)|CDKN2A:c.172C>T (p.Arg58Ter)|TP53:c.818G>A (p.Arg273His)|		4,2	

60	51	Male	FANCA:c.3094C>T (p.Gln1032Ter)|KRAS:c.35G>A (p.Gly12Asp)|STK11:c.923G>A (p.Trp308Ter)|STK11:c.923G>A (p.Trp308Ter)|TP53:c.310C>T (p.Gln104Ter)|Microsatellite Instability (MSI):STABLE (MSS)|PD-L1 expression:negative <1%|	negative|<1%	fail	STABLE (MSS)
61	73	Female	KRAS:c.35G>T (p.Gly12Val)|PIK3CA:c.3139C>T (p.His1047Tyr)|		20	
62	39	Male	PALB2:c.1140_1143delTCTT (p.Ser380fs)|		0,9	
63	49	Male	TP53:c.916C>T (p.Arg306Ter)|		9,4	
64	64	Female	KRAS:c.35G>T (p.Gly12Val)|		29,6	
65	65	Female	KRAS:c.35G>A (p.Gly12Asp)|TP53:c.731G>T (p.Gly244Val)|Microsatellite Instability (MSI):STABLE (MSS)|PD-L1 expression:positive TC 15%, IC 1%|	positive|TC 15%, IC 1%	4,33	STABLE (MSS)
66	58	Male	KRAS:c.35G>T (p.Gly12Val)|CDKN2A:c.7G>T (p.Glu27Ter)|TP53:c.646G (p.Val216Leu)|Microsatellite Instability (MSI):STABLE (MSS)|PD-L1 expression:positive TC<1%, IC<1%|	positive|TC<1%, IC<1%	10,81	STABLE (MSS)
67	73	Male			4,05	

68	59	Female	KRAS:c.35G>A (p.Gly12Asp)|SMAD4:c.1576G>T (p.Glu526Ter)|TP53:c.733G>A (p.Gly245Ser)|		18,59	
69	71	Male	KRAS:c.35G>A (p.Gly12Asp)|TP53:c.524G>A (p.Arg175His)|Microsatellite Instability (MSI):STABLE (MSS)|PD-L1 expression:positive TC<1%, IC 5%|	positive|TC<1%, IC 5%	4,02	STABLE (MSS)
70	61	Male	MLH1:c.1292_1293insC (p.Met431fs)|FBXW7:c.1629_1630delAG (p.Arg543fs)|FBXW7:c.1099C>T (p.Arg367Ter)|CDKN2B:c.244C>T (p.Arg82Ter)|			
71	71	Male	KRAS:c.34G>C (p.Gly12Arg)|		6,13	
72	54	Male	KRAS:c.38G>A (p.Gly13Asp)|TP53:c.742C>T (p.Arg248Trp)|Microsatellite Instability (MSI):STABLE (MSS)|PD-L1 expression:positive TC>10%, IC>50%|	positive|TC>10%, IC>50%	3,65	STABLE (MSS)
73	41	Male	KRAS:c.35G>A (p.Gly12Asp)|			
74	61	Female	KRAS:c.34G>C (p.Gly12Arg)|			
75	80	Male	KRAS:c.35G>A (p.Gly12Asp)|			
76	57	Male	KRAS:c.183A>C (p.Gln61His)|			

77	73	Male	KRAS:c.35G>T (p.Gly12Val)|TP53:c.577C>T (p.His193Tyr)|Microsatellite Instability (MSI):STABLE (MSS)|PD-L1 expression:positive TC <1%, IC 1%|	positive|TC <1%, IC 1%	8,32	STABLE (MSS)
78	70	Male	KRAS:c.35G>T (p.Gly12Val)|Microsatellite Instability (MSI):STABLE (MSS)|PD-L1 expression:negative IC<1%|	negative|IC<1%	27,31	STABLE (MSS)
79	84	Female	NF2:c.1331C>A (p.Ser444Ter)|NF2:c.1294G>T (p.Glu432Ter)|TP53:c.817C>T (p.Arg273Cys)|MYC:Amplification|		25,45	
80	69	Male	KRAS:c.35G>A (p.Gly12Asp)|TP53:c.480_496delGGCTCTAAGGT (p.Met160fs)|Microsatellite Instability (MSI):STABLE (MSS)|PD-L1 expression:positive TC 10%, IC<1%|	positive|TC 10%, IC<1%	5,18	STABLE (MSS)
81	58	Male	KRAS:c.35G>A (p.Gly12Asp)|TP53:c.839G>C (p.Arg280Thr)|		12,85	
82	57	Male	Microsatellite Instability (MSI):STABLE (MSS)|PD-L1 expression:negative ncelled|	negative|ncelled	2,57	STABLE (MSS)
83	57	Female				
84	61	Male	KRAS:c.34G>T (p.Gly12Cys)|TP53:c.524G>A (p.Arg175His)|			

85	55	Male	KRAS:c.35G>A (p.Gly12Asp)|PIK3CA:c.1633G>A (p.Glu545Lys)|TP53:c.524G>A (p.Arg175His)|			
86	56	Male	KRAS:c.35G>A (p.Gly12Asp)|MET fusion:MET(13)-MET(15)|Microsatellite Instability (MSI):STABLE (MSS)|PD-L1 expression:positive TC<1%, IC<1%|	negative| TC<1%, IC<1%	fail	STABLE (MSS)
87	67	Male	KRAS:c.35G>A (p.Gly12Asp)|Microsatellite Instability (MSI):STABLE (MSS)|PD-L1 expression:negative TC<1%, IC<1%|	negative| TC<1%, IC<1%	9,35	STABLE (MSS)
88	55	Male	KRAS:c.35G>A (p.Gly12Asp)|Microsatellite Instability (MSI):STABLE (MSS)|PD-L1 expression:positive TC 1%, IC<1%|	positive|TC 1%, IC<1%	2,54	STABLE (MSS)
89	64	Female	KRAS:c.35G>A (p.Gly12Asp)|Microsatellite Instability (MSI):STABLE (MSS)|PD-L1 expression:positive TC<1%, IC 20%|	positive|TC<1%, IC 20%	1,72	STABLE (MSS)
90	63	Male	KRAS:c.35G>T (p.Gly12Val)|TP53:c.566_567insCCCT (p.Gln192fs)|			
91	64	Female	KRAS:c.35G>A (p.Gly12Asp)|PIK3CA:c.331A>G (p.Lys111Glu)|CDKN2A:c.172C>T (p.Arg58Ter)|TP53:c.472C>G (p.Arg158Gly)|		3,97	

92	71	Female	KRAS:c.34G>C (p.Gly12Arg)|CDKN2A:c.47_50delTGGC (p.Leu16fs)|TP53:c.743G>A (p.Arg248Gln)|Microsatellite Instability (MSI):STABLE (MSS)|PD-L1 expression:positive TC<1%, IC 20%|	positive|TC<1%, IC 20%	11,14	STABLE (MSS)
93	73	Female	KRAS:c.35G>T (p.Gly12Val)|			
94	56	Male	KRAS:c.35G>A (p.Gly12Asp)|TP53:c.578A>G (p.His193Arg)|Microsatellite Instability (MSI):STABLE (MSS)|PD-L1 expression:positive TC<1%, IC 25%|	positive|TC<1%, IC 25%	2,57	STABLE (MSS)
95	44	Female	KRAS:c.35G>A (p.Gly12Asp)|Microsatellite Instability (MSI):STABLE (MSS)|PD-L1 expression:positive TC1%, IC<1%|	positive|TC1%, IC<1%	8,03	STABLE (MSS)
96	45	Male	Microsatellite Instability (MSI):STABLE (MSS)|PD-L1 expression:negative TC<1%, IC<1%|	negative| TC<1%, IC<1%	18,38	STABLE (MSS)
97	73	Male	KRAS:c.35G>T (p.Gly12Val)|			
98	68	Male	KRAS:c.183A>C (p.Gln61His)|TP53:c.524G>A (p.Arg175His)|ERBB2:Amplification|			
99	61	Male			14,63	
100	72	Male	KRAS:c.35G>T (p.Gly12Val)|CDKN2A:c.135_138delTCGG (p.Arg46fs)|			
101	72	Female	KRAS:c.34G>C (p.Gly12Arg)|			

102	65	Male	KRAS:c.35G>A (p.Gly12Asp)|ARID1A:c.6466delA (p.Ser2156fs)|TP53:c.742C>T (p.Arg248Trp)|			
103	51	Male	KRAS:c.35G>T (p.Gly12Val)|TP53:c.526T>A (-)|:|:|			
104	57	Female	KRAS:c.35G>A (p.Gly12Asp)|CDKN2A:c.247C>T (p.His83Tyr)|TP53:c.226_227insG (p.Ala76fs)|Microsatellite Instability (MSI):STABLE (MSS)|PD-L1 expression:positive TC 10%, IC 1%|	positive|TC 10%, IC 1%	6,12	STABLE (MSS)
105	53	Female	NBN:c.657_661delACAAA (p.Lys219Asnfs*16)|			
106	56	Female	KRAS:c.35G>T (p.Gly12Val)|STK11:c.464+1G>A|STK11:c.464+1G>A|			
107	80	Male	KRAS:c.35G>A (p.Gly12Asp)|TP53:c.586C>T (p.Arg196Ter)|TP53:c.173delC (p.Pro58fs)|			
108	64	Male	KRAS:c.35G>T (p.Gly12Val)|TP53:c.577C>T (p.His193Tyr)|			
109	67	Female	KRAS:c.35G>T (p.Gly12Val)|TP53:c.832C>T (p.Pro278Ser)|			
110	54	Male	KRAS:c.34G>C (p.Gly12Arg)|KRAS:amplification|MYC:amplification|CCND3:amplification|			
111	60	Male	KRAS:c.35G>A (p.Gly12Asp)|TP53: c.833C>T (p.Pro278Leu)|			
112	66	MAle	KRAS:c.35G>A (p.Gly12Asp)|GNAS:c.602G>A (p.Arg201His)|			

113	64	Male				
114	62	Female	KRAS:c.35G>T (p.Gly12Val)|			
115	54	Male	KRAS:c.35G>T (p.Gly12Val)|TP53:c.524G>A (p.Arg175His)|			
116	51	Male	KRAS:c.35G>T (p.Gly12Val)|TP53:c.451C>T (p.Pro151Ser)|Microsatellite Instability (MSI):MSS|PD-L1 expression:negative TC<1%, IC<1%|	negative| TC<1%, IC<1%	3,81	MSS
117	-	Female	KRAS:c.34G>C (p.Gly12Arg)|TP53:c.818G>T (p.Arg273Leu)|Microsatellite Instability (MSI):MSS|		5,79	MSS
118	53	Female	KRAS:c.35G>A (p.Gly12Asp)|HNF1A:c.864delG (p.Pro291GlnfsTer51)|TP53:c.78delT (p.Pro27LeufsTer17)|Microsatellite Instability (MSI):MSS|PD-L1 expression:negative TC<1%, IC<1%|ERCC1:Low expression|TYMS:Low expression|UGT1A1:HETEROZYGOUS (6/7)|DPD:WT|	negative| TC<1%, IC<1%	6,1	MSS
119	68	Female	KRAS:c.34G>C (p.Gly12Arg)|TP53:c.332T>A (p.Leu111Gln)|		7,26	

<table>
<tr><td>120</td><td>58</td><td>Male</td><td>KRAS:c.35G>A (p.Gly12Asp)|Microsatellite Instability (MSI):MSS|PD-L1 expression:- TC 30%, IC 2%|Tumor Mutational Burden (TMB):5,08 Muts/MB|</td><td>-|TC 30%, IC 2%</td><td>5,08</td><td>MSS</td></tr>
<tr><td>121</td><td>-</td><td>Male</td><td>ALK:c.3452C>T (p.Thr1151Met)|ATM:c.7916delA (p.Lys2639ArgfsTer7)|FGFR4:c.749_768delinsGGA (p.Ile250Argfs*57)|</td><td></td><td></td><td></td></tr>
<tr><td>122</td><td>78</td><td>Female</td><td>KRAS:c.35G>A (p.Gly12Asp)|Tumor Mutational Burden (TMB):14,58 Muts/MB|</td><td></td><td>14,58</td><td></td></tr>
<tr><td>123</td><td>32</td><td>Male</td><td>KRAS:c.35G>T (p.Gly12Val)|NF2:c.1321delG (p.Ala441LeufsTer14)|Microsatellite Instability (MSI):MSS|Tumor Mutational Burden (TMB):2,9 Muts/MB|</td><td></td><td>2,9</td><td>MSS</td></tr>
<tr><td>124</td><td>62</td><td>Male</td><td>KRAS:c.35G>A (p.Gly12Asp)|MUTYH:c.536A>G (p.Tyr179Cys)|Microsatellite Instability (MSI):MSS|Tumor Mutational Burden (TMB):2.03 Muts/MB|</td><td></td><td>2,03</td><td>MSS</td></tr>
<tr><td>125</td><td>53</td><td>Male</td><td>KRAS:c.34G>C (p.Gly12Arg)|TP53:c.614A>G (p.Tyr205Cys)|Microsatellite Instability (MSI):MSS|Tumor Mutational Burden (TMB):3.86 Muts/MB|</td><td></td><td>3,86</td><td>MSS</td></tr>
</table>

<table>
<tr><td>126</td><td>66</td><td>Male</td><td>KRAS:c.35G>T (p.Gly12Val)|TP53:c.742C>T (p.Arg248Trp)|Microsatellite Instability (MSI):MSS|PD-L1 expression:negative TC<1%, IC<1%|Tumor Mutational Burden (TMB):13.03 Muts/MB|</td><td>negative| TC<1%, IC<1%</td><td>13,03</td><td>MSS</td></tr>
<tr><td>127</td><td>77</td><td>Male</td><td>ATM:c.3673C>T (p.Gln1225Ter)|BRCA2:c.8174G>A (p.Trp2725Ter)|CDKN2A:c.130delT (p.Tyr44ThrfsTer9)|PALB2:c.2761C>T (p.Gln921Ter)|POLE:c.3038G>A (p.Trp1013Ter)|STK11:c.1111C>T (p.Gln371Ter)|STK11:c.1111C>T (p.Gln371Ter)|RAD50:c.2821C>T (p.Gln941Ter)|</td><td></td><td></td><td></td></tr>
<tr><td>128</td><td>65</td><td>Male</td><td>KRAS:c.183A>C (p.Gln61His)|</td><td></td><td></td><td></td></tr>
<tr><td>129</td><td>70</td><td>Female</td><td>KRAS:c.35G>T (p.Gly12Val)|TP53:c.646G>A (p.Val216Met)|MAP2K7:c.745delA (p.Ile249SerfsTer162)|AR:amplification|CCNE1:amplification|Microsatellite Instability (MSI):MSS|PD-L1 expression:negative TC<1%, IC<1%|Tumor Mutational Burden (TMB):5.82 Muts/MB|</td><td>negative| TC<1%, IC<1%</td><td>5,82</td><td>MSS</td></tr>
<tr><td>130</td><td>37</td><td>Female</td><td>KRAS:c.35G>A (p.Gly12Asp)|</td><td></td><td></td><td></td></tr>
</table>

131	66	Male	PALB2:c.2747_2748+4delAGGTAA|Microsatellite Instability (MSI):MSS|PD-L1 expression:negative TC <1%, IC<1% |Tumor Mutational Burden (TMB):3.84 Muts/MB|	negative| TC <1%, IC<1%	3,84	MSS
132	70	Male	SF3B1:c.1998G>C (p.Lys666Asn)|DSC3:c.2503C>T (p.Arg835Ter)|Microsatellite Instability (MSI):MSS|PD-L1 expression: TC<1%, IC<1%|Tumor Mutational Burden (TMB):9.4 Muts/MB|	unknown negative| TC<1%, IC<1%	9,4	MSS
133	72	Female	KRAS:c.35G>A (p.Gly12Asp)|CDKN2A:c.381_435delACGGTACCTGCGCGCGGCTGCGGGGGCACCAGAGGCAGTAACCATGCCCGCATA (p.Arg128MetfsTer9)|Microsatellite Instability (MSI):MSS|PD-L1 expression:negative TC<1%, IC<1%|Tumor Mutational Burden (TMB):9.59 Muts/MB|ERCC1:Low expression|TYMS:Low expression|	negative| TC<1%, IC<1%	9,59	MSS
134	83	Male	MET:amplification|			
135	65	Male	KRAS:c.35G>A (p.Gly12Asp)|GNAS:c.601C>T (p.Arg201Cys)|PD-L1 expression: TC<1%, IC=2|Tumor Mutational Burden (TMB):6.9 Muts/MB|	unknown positive|TC<1%, IC=2	6,9	

136	52	Male	Microsatellite Instability (MSI):MSS|PD-L1 expression:negative TC<1% IC<1%|Tumor Mutational Burden (TMB):9.52 Muts/MB|	negative| TC<1% IC<1%	9,52	MSS
137	79	Female	KRAS:c.772G>A (p.Glu258Lys)|TP53:c.35G>A (p.Gly12Asp)|Microsatellite Instability (MSI):MSS|PD-L1 expression:positive TC>50%, IC<1%|Tumor Mutational Burden (TMB):4,78 Muts/MB|ERCC1:Low expression|TYMS:Low expression|	positive|TC>50%, IC<1%	4,78	MSS
138	77	Male	Microsatellite Instability (MSI):MSS|PD-L1 expression:negative TC<1%, IC<1%|Tumor Mutational Burden (TMB):3,87 Muts/MB|ERCC1:Low expression|TYMS:Low expression|	negative| TC<1%, IC<1%	3,87	MSS
139	57	Female	KRAS:c.35G>A (p.Gly12Asp)|TP53:c.702C>G (p.Tyr234Ter)|Microsatellite Instability (MSI):MSS|PD-L1 expression:positive TC>50%, IC<1%|Tumor Mutational Burden (TMB):4,92 Muts/MB|ERCC1:High expression|TYMS:Low expression|	positive|TC>50%, IC<1%	4,92	MSS
140	51	Male	KRAS:c.35G>A (p.Gly12Asp)|TP53:c.856G>A (p.Glu286Lys)|			

141	81	Female	KRAS:c.35G>T (p.Gly12Val)|Microsatellite Instability (MSI):MSS|PD-L1 expression:negative TC<1% IC<1%|Tumor Mutational Burden (TMB):3,82 Muts/MB|	negative| TC<1% IC<1%	3,82	MSS
142	50	Male	Microsatellite Instability (MSI):MSS|PD-L1 expression:negative TC<1% IC<1%|Tumor Mutational Burden (TMB):3,81 Muts/MB|ERCC1:High expression|TYMS:Low expression|	negative| TC<1% IC<1%	3,81	MSS
143	63	Female	KRAS:c.35G>A (p.Gly12Asp)|MDM2:amplification|Tumor Mutational Burden (TMB):5,94 Muts/MB|		5,94	
144	51	Female	KRAS:c.35G>A (p.Gly12Asp)|TP53:c.586C>T (p.Arg196Ter)|Microsatellite Instability (MSI):MSS|PD-L1 expression:negative TC<1% IC<1%|Tumor Mutational Burden (TMB):21,85 Muts/MB|ERCC1:Low expression|TYMS:Low expression|	negative| TC<1% IC<1%	21,85	MSS
145	58	Male	KRAS:c.35G>T (p.Gly12Val)|CDKN2A:c.138delG (p.Arg47GlyfsTer6)|TP53:c.827C>A (p.Ala276Asp)|			

146	63	Female	KRAS:c.34G>C (p.Gly12Arg)|Microsatellite Instability (MSI):MSS|PD-L1 expression:negative TC<1% IC<1%|Tumor Mutational Burden (TMB):2,92 Muts/MB|ERCC1:High expression|TYMS:Low expression|	negative| TC<1% IC<1%	2,92	MSS
147	71	Female	KRAS:c.35G>T (p.Gly12Val)|TP53:c.796G>T (p.Gly266Ter)|ATR:c.5605C>T (p.Gln1869Ter)|Microsatellite Instability (MSI):MSS|PD-L1 expression:positive TC 30%, IC<1%|Tumor Mutational Burden (TMB): Muts/MB|ERCC1:Low expression|TYMS:Low expression|	positive|TC 30%, IC<1%		MSS
148	64	Male	KRAS:c.35G>A (p.Gly12Asp)|Microsatellite Instability (MSI):MSS|PD-L1 expression:negative TC<1% IC<1%|Tumor Mutational Burden (TMB):6,77 Muts/MB|	negative| TC<1% IC<1%	6,77	MSS
149	66	Male	KRAS:c.35G>T (p.Gly12Val)|TP53:c.743G>A (p.Arg248Gln)|Microsatellite Instability (MSI):MSS|PD-L1 expression:positive TC 2%, IC<1%|Tumor Mutational Burden (TMB):5,76 Muts/MB|	positive|TC 2%, IC<1%	5,76	MSS

150	49	Female	KRAS:c.34G>C (p.Gly12Arg)|Microsatellite Instability (MSI):MSS|PD-L1 expression:positive TC 30% IC 1%|Tumor Mutational Burden (TMB):12,39 Muts/MB|	positive|TC 30% IC 1%	12,39	MSS
151	62	Female	KRAS:c.35G>A (p.Gly12Asp)|TP53:c.919+2T>G (splicing)|Microsatellite Instability (MSI):MSS|PD-L1 expression:positive TC 30%, IC<1%|Tumor Mutational Burden (TMB):1,91 Muts/MB|	positive|TC 30%, IC<1%	1,91	MSS
152	70	Female	KRAS:c.182A>G (p.Gln61Arg)|		7,01	
153	72	Female	KRAS:c.35G>T (p.Gly12Val)|Microsatellite Instability (MSI):MSS|PD-L1 expression:negative TC<1% IC<1%|Tumor Mutational Burden (TMB):14,29 Muts/MB|ERCC1:Low expression|TYMS:Low expression|	negative| TC<1% IC<1%	14,29	MSS
154	50	Male	KRAS:c.35G>A (p.Gly12Asp)|CDKN2A:c.238C>T (p.Arg80Ter)|TP53:c.742C>T (p.Arg248Trp)|Microsatellite Instability (MSI):MSS|PD-L1 expression:positive TC<1%, IC 5%|Tumor Mutational Burden (TMB):4,78 Muts/MB|	positive|TC<1%, IC 5%	4,78	MSS

155	57	Male	KRAS:c.34G>C (p.Gly12Arg)|TP53:c.742C>T (p.Arg248Trp)|Microsatellite Instability (MSI):MSS|PD-L1 expression:positive TC<1 IC2%|Tumor Mutational Burden (TMB):0,95 Muts/MB|	positive|TC<1 IC2%	0,95	MSS
156	74	Male	KRAS:c.183A>C (p.Gln61His)|NOTCH1:c.2047G>A (p.Ala683Thr)|Microsatellite Instability (MSI):MSS|PD-L1 expression:negative TC<1% IC<1%|Tumor Mutational Burden (TMB):3,89 Muts/MB|	negative| TC<1% IC<1%	3,89	MSS
157	55	Male	KRAS:c.35G>T (p.Gly12Val)|TP53:c.421T>C (p.Cys141Arg)|CDKN2A:c.262G>T (p.Glu88Ter)|SMAD4:c.719_720insT (p.Ala241SerfsTer23)|Microsatellite Instability (MSI):MSS|PD-L1 expression:negative TC<1%, IC<1%|Tumor Mutational Burden (TMB):7,71 Muts/MB|	negative| TC<1%, IC<1%	7,71	MSS
158	57	Female	KRAS:c.35G>T (p.Gly12Val)|TP53:c.524G>A (p.Arg175His)|KRAS:amplification|Microsatellite Instability (MSI):MSS|PD-L1 expression:negative TC<1%, IC<1%|Tumor Mutational Burden (TMB):1,89 Muts/MB|	negative| TC<1%, IC<1%	1,89	MSS

159	74	Female	KRAS:c.34G>C (p.Gly12Arg)|RSPO2 fusion:EIF3E(1) - RSPO2(2)|Microsatellite Instability (MSI):MSS|PD-L1 expression:negative TC<1% IC<1%|Tumor Mutational Burden (TMB):5,87 Muts/MB|	negative| TC<1% IC<1%	5,87	MSS
160	61	Male	KRAS:c.35G>A (p.Gly12Asp)|TP53:c.402T>G (p.Phe134Leu)|Microsatellite Instability (MSI):MSS|PD-L1 expression:positive TC<1%, IC 30%|Tumor Mutational Burden (TMB):4,8 Muts/MB|	positive|TC<1%, IC 30%	4,8	MSS
161	63	Male	KRAS:c.35G>T (p.Gly12Val)|TP53:c.599+2T>G ()|Microsatellite Instability (MSI):MSS|PD-L1 expression:positive TC 2%, IC<1%|Tumor Mutational Burden (TMB):3,84 Muts/MB|	positive|TC 2%, IC<1%	3,84	MSS
162	41	Male	KRAS:c.34G>C (p.Gly12Arg)|TP53:c.856G>A (p.Glu286Lys)|SMAD4:c.1333C>T (p.Arg445Ter)|Microsatellite Instability (MSI):MSS|PD-L1 expression:negative TC<1% IC<1%|Tumor Mutational Burden (TMB):6,76 Muts/MB|	negative| TC<1% IC<1%	6,76	MSS
163	78	Male	ATM:c.5980A>T (p.Lys1994Ter)|KRAS:c.34G>C (p.Gly12Arg)|			

164	67	Female	KRAS:c.35G>T (p.Gly12Val)|RNF43:c.1418delG (p.Gly473AlafsTer29)|TP53:c.586C>T (p.Arg196Ter)|Microsatellite Instability (MSI):MSS|PD-L1 expression:positive TC<1%, IC 10%|Tumor Mutational Burden (TMB):5,78 Muts/MB|	positive|TC<1%, IC 10%	5,78	MSS
165	62	Male	KRAS:c.35G>A (p.Gly12Asp)|Microsatellite Instability (MSI):MSS|PD-L1 expression:positive TC 10%, IC<1%|Tumor Mutational Burden (TMB):7,73 Muts/MB|ERCC1:Low expression|TYMS:Low expression|	positive|TC 10%, IC<1%	7,73	MSS
166	69	Male	KRAS:c.34G>C (p.Gly12Arg)|TP53:c.817C>T (p.Arg273Cys)|Microsatellite Instability (MSI):MSS|PD-L1 expression:positive TC 20%, IC 10%|Tumor Mutational Burden (TMB):4,88 Muts/MB|	positive|TC 20%, IC 10%	4,88	MSS
167	60	Male	KRAS:c.35G>A (p.Gly12Asp)|Microsatellite Instability (MSI):MSS|PD-L1 expression:positive TC 5% IC 15%|Tumor Mutational Burden (TMB):0,96 Muts/MB|	positive|TC 5% IC 15%	0,96	MSS

168	73	Male	KRAS:c.183A>T (p.Gln61His)|Microsatellite Instability (MSI):MSS|PD-L1 expression:negative TC<1%, IC<1%|Tumor Mutational Burden (TMB):4,83 Muts/MB|ERCC1:Low expression|TYMS:Low expression|UGT1A1:WT (6/6)|DPD:WT|	negative| TC<1%, IC<1%	4,83	MSS
169	82	Female	BRCA1:c.5251C>T (p.Arg1751Ter)|KRAS:c.35G>A (p.Gly12Asp)|TP53:c.687T>A (p.Cys229Ter)|RASA2:c.2023delC (p.Gln675LysfsTer2)|AR:amplification|SLCO1B3:amplification|Microsatellite Instability (MSI):MSS|PD-L1 expression:positive TC 10% IC<1%|Tumor Mutational Burden (TMB):6,7 Muts/MB|	positive|TC 10% IC<1%	6,7	MSS
170	58	Male	KRAS:c.35G>A (p.Gly12Asp)|STK11:c.180C>G (p.Tyr60Ter)|STK11:c.180C>G (p.Tyr60Ter)|Microsatellite Instability (MSI):MSS|PD-L1 expression:negative TC<1%, IC<1%|Tumor Mutational Burden (TMB):4,29 Muts/MB|	negative| TC<1%, IC<1%	4,29	MSS

171	80	Male	KRAS:c.34G>C (p.Gly12Arg)|TP53:c.841G>A (p.Asp281Asn)|Microsatellite Instability (MSI):MSS|PD-L1 expression:negative TC<1%, IC<1%|Tumor Mutational Burden (TMB):1,93 Muts/MB|ERCC1:Low expression|TYMS:Low expression|UGT1A1:HETEROZYGOUS (6/7)|DPD:WT|	negative| TC<1%, IC<1%	1,93	MSS
172	71	Male	CDKN2A:c.186_187insG (p.Leu63AlafsTer57)|ATM:c.9023G>A (p.Arg3008His)|KRAS:c.35G>A (p.Gly12Asp)|RNF43:c.714G>A (p.Trp238Ter)|CDK2:amplification|			
173	64	Male	KRAS:c.35G>A (p.Gly12Asp)|TP53:c.524G>A (p.Arg175His)|CDKN2A:c.172C>T (p.Arg58Ter)|Microsatellite Instability (MSI):MSS|PD-L1 expression:negative TC<1%, IC<1%|Tumor Mutational Burden (TMB):4,84 Muts/MB|ERCC1:Low expression|TYMS:Low expression|UGT1A1:WT (6/6)|DPD:WT|	negative| TC<1%, IC<1%	4,84	MSS

174	51	Male	KRAS:c.35G>A (p.Gly12Asp)|Microsatellite Instability (MSI):MSS|PD-L1 expression:negative TC<1%, IC<1%|Tumor Mutational Burden (TMB):0,96 Muts/MB|	negative| TC<1%, IC<1%	0,96	MSS
175	58	Female	KRAS:c.35G>A (p.Gly12Asp)|TP53:c.818G>A (p.Arg273His)|Microsatellite Instability (MSI):MSS|Tumor Mutational Burden (TMB):12,7 Muts/MB|		12,7	MSS
176	65	Male	KRAS:c.35G>A (p.Gly12Asp)|SMAD4:c.353C>T (p.Ala118Val)|RSPO2 fusion:EIF3E(1) - RSPO2(2)|Microsatellite Instability (MSI):MSS|PD-L1 expression:negative TC<1%, IC<1%|Tumor Mutational Burden (TMB):6,87 Muts/MB|	negative| TC<1%, IC<1%	6,87	MSS
177	57	Male	KRAS:c.35G>T (p.Gly12Val)|TP53:c.743G>A (p.Arg248Gln)|Microsatellite Instability (MSI):MSS|PD-L1 expression:negative TC<1%, IC<1%|Tumor Mutational Burden (TMB):4,79 Muts/MB|ERCC1:Low expression|TYMS:Low expression|	negative| TC<1%, IC<1%	4,79	MSS
178	54	Female	KRAS:c.35G>T (p.Gly12Val)|TP53:c.916C>T (p.Arg306Ter)|CDKN2A:c.233_234insT (p.Thr79fs)|			

<table>
<tr><td>179</td><td>63</td><td>Female</td><td>KRAS:c.35G>A (p.Gly12Asp)|TP53:c.524G>A (p.Arg175His)|Microsatellite Instability (MSI):MSS|PD-L1 expression:negative TC<1%, IC<1%|Tumor Mutational Burden (TMB):32,89 Muts/MB|ERCC1:Low expression|TYMS:Low expression|</td><td>negative| TC<1%, IC<1%</td><td>32,89</td><td>MSS</td></tr>
<tr><td>180</td><td>66</td><td>Female</td><td>KRAS:c.34G>C (p.Gly12Arg)|SMAD4:c.175delA (p.Thr59GlnfsTer3)|Microsatellite Instability (MSI):MSS|PD-L1 expression:negative TC<1% IC<1%|Tumor Mutational Burden (TMB):2,86 Muts/MB|</td><td>negative| TC<1% IC<1%</td><td>2,86</td><td>MSS</td></tr>
<tr><td>181</td><td>71</td><td>Male</td><td>KRAS:c.34G>C (p.Gly12Arg)|Microsatellite Instability (MSI):MSS|PD-L1 expression:negative TC<1% IC<1%|Tumor Mutational Burden (TMB):5,72 Muts/MB|</td><td>negative| TC<1% IC<1%</td><td>5,72</td><td>MSS</td></tr>
<tr><td>182</td><td>64</td><td>Female</td><td>KRAS:c.34G>C (p.Gly12Arg)|TP53:c.701A>G (p.Tyr234Cys)|</td><td></td><td></td><td></td></tr>
<tr><td>183</td><td>59</td><td>Female</td><td>KRAS:c.35G>A (p.Gly12Asp)|TP53:c.637C>T (p.Arg213Ter)|AR:amplification|Microsatellite Instability (MSI):MSS|PD-L1 expression:negative TC<1% IC<1%|Tumor Mutational Burden (TMB):3,81 Muts/MB|</td><td>negative| TC<1% IC<1%</td><td>3,81</td><td>MSS</td></tr>
</table>

184	62	Female	KRAS:c.35G>A (p.Gly12Asp)|Microsatellite Instability (MSI):MSS|PD-L1 expression:positive TC<1%, IC 2%|Tumor Mutational Burden (TMB):10,29 Muts/MB|	positive|TC<1%, IC 2%	10,29	MSS
185	60	Male	KRAS:c.35G>A (p.Gly12Asp)|CDKN2A:c.47_50delTGGC (p.Leu16ProfsTer9)|TP53:c.833C>T (p.Pro278Leu)|Microsatellite Instability (MSI):MSS|PD-L1 expression:positive TC<1%, IC 15%|Tumor Mutational Burden (TMB):7,81 Muts/MB|	positive|TC<1%, IC 15%	7,81	MSS
186	43	Female				
187	51	Male	Microsatellite Instability (MSI):-|PD-L1 expression:negative TC<1% IC<1%|Tumor Mutational Burden (TMB): Muts/MB|	negative| TC<1% IC<1%		-

188	66	Female	KRAS:c.35G>T (p.Gly12Val)|TP53:c.659A>G (p.Tyr220Cys)|GNAS:c.602G>A (p.Arg201His)|STED2:c.2324_2325delTT (p.Val775GlufsTer5)|Microsatellite Instability (MSI):MSS|PD-L1 expression:negative TC<1% IC<1%|Tumor Mutational Burden (TMB):2,9 Muts/MB|ERCC1:Low expression|TYMS:Low expression|	negative| TC<1% IC<1%	2,9	MSS
189	36	Female	Microsatellite Instability (MSI):MSS|PD-L1 expression:negative |Tumor Mutational Burden (TMB):0,96 Muts/MB|	negative|	0,96	MSS
190	58	Female	KRAS:c.34G>C (p.Gly12Arg)|Microsatellite Instability (MSI):MSS|PD-L1 expression:negative TC<1% IC<1%|Tumor Mutational Burden (TMB):1,92 Muts/MB|ERCC1:Low expression|TYMS:Low expression|UGT1A1:HETEROZYGOUS (6/7)|DPD:WT|	negative| TC<1% IC<1%	1,92	MSS

191	55	Male	BRCA2:c.3374_3375delTT (p.Phe1125Ter)|CDKN2A:c.131_132insA (p.Tyr44Ter)|KRAS:c.35G>A (p.Gly12Asp)|PIK3CA:c.1358A>G (p.Glu453Gly)|TP53:c.817C>T (p.Arg273Cys)|FBXW7:c.1393C>T (p.Arg465Cys)|Microsatellite Instability (MSI):MSS|PD-L1 expression:negative TC<1%, IC<1%|Tumor Mutational Burden (TMB):7,33 Muts/MB|ERCC1:Low expression|TYMS:Low expression|	negative| TC<1%, IC<1%	7,33	MSS
192	60	Female	KRAS:c.35G>T (p.Gly12Val)|TP53:c.646G>A (p.Val216Met)|Microsatellite Instability (MSI):MSS|PD-L1 expression:negative TC<1% IC<1%|Tumor Mutational Burden (TMB):4,12 Muts/MB|ERCC1:High expression|TYMS:Low expression|	negative| TC<1% IC<1%	4,12	MSS
193	79	Female	KRAS:c.34G>A (p.Gly12Ser)|Microsatellite Instability (MSI):MSS|PD-L1 expression:negative TC<1%, IC<1%|Tumor Mutational Burden (TMB):4,82 Muts/MB|ERCC1:Low expression|TYMS:Low expression|	negative| TC<1%, IC<1%	4,82	MSS

194	68	Female	KRAS:c.35G>A (p.Gly12Asp)|TP53:c.524G>A (p.Arg175His)|Microsatellite Instability (MSI):MSS|PD-L1 expression:negative TC<1%, IC<1%|Tumor Mutational Burden (TMB):1,98 Muts/MB|	negative| TC<1%, IC<1%	1,98	MSS
195	65	Male	TP53:c.997delC (p.Arg333ValfsTer12)|			
196	49	Female	ATM:c.8545C>T (p.Arg2849Ter)|ATM:c.1564G>T (p.Glu522Ter)|KRAS:c.35G>A (p.Gly12Asp)|SMAD4:c.403C>T (p.Arg135Ter)|RNF43:c.1436_1437delTG (p.Val479GlyfsTer25)|PIK3CA:c.263G>A (p.Arg88Gln)|Microsatellite Instability (MSI):MSS|PD-L1 expression:positive TC 50%,IC 5%|Tumor Mutational Burden (TMB):9,55 Muts/MB|ERCC1:Low expression|TYMS:Low expression|	positive|TC 50%,IC 5%	9,55	MSS
197	73	Female	KRAS:c.35G>A (p.Gly12Asp)|TP53:c.743G>A (p.Arg248Gln)|Microsatellite Instability (MSI):MSS|PD-L1 expression:negative TC<1% IC<1%|Tumor Mutational Burden (TMB):4,82 Muts/MB|ERCC1:Low expression|TYMS:Low expression|	negative| TC<1% IC<1%	4,82	MSS

198	60	Male	KRAS:c.34G>C (p.Gly12Arg)|TP53:c.742C>T (p.Arg248Trp)|MYC:amplification|Microsatellite Instability (MSI):MSS|PD-L1 expression:negative TC<1% IC<1%|Tumor Mutational Burden (TMB):2,98 Muts/MB|	negative| TC<1% IC<1%	2,98	MSS
199	60	Female	KRAS:c.34G>C (p.Gly12Arg)|SMAD4:c.362T>A (p.Leu121Ter)|TP53:c.817C>T (p.Arg273Cys)|FGF23:amplification|CCND2:amplification|Microsatellite Instability (MSI):MSS|Tumor Mutational Burden (TMB):8,61 Muts/MB|		8,61	MSS
200	82	Female	KRAS:c.35G>T (p.Gly12Val)|TP53:c.710T>A (p.Met237Lys)|Microsatellite Instability (MSI):MSS|PD-L1 expression:negative TC<1% IC<1%|Tumor Mutational Burden (TMB):3,99 Muts/MB|ERCC1:High expression|TYMS:Low expression|UGT1A1:WT (6/6)|DPD:WT|	negative| TC<1% IC<1%	3,99	MSS

201	43	Female	TP53:c.679_690delTCTGACTGTACC (p.Ser227_Thr230del)|MET:amplification|MRE11:amplification|Microsatellite Instability (MSI):MSS|PD-L1 expression:negative TC<1% IC<1%|Tumor Mutational Burden (TMB):9,8 Muts/MB|ERCC1:High expression|TYMS:Low expression|	negative| TC<1% IC<1%	9,8	MSS
202	77	Male	Microsatellite Instability (MSI):MSS|PD-L1 expression:negative TC<1% IC<1%|Tumor Mutational Burden (TMB):1,96 Muts/MB|ERCC1:Low expression|TYMS:Low expression|	negative| TC<1% IC<1%	1,96	MSS
203	81	Male	KRAS:c.35G>T (p.Gly12Val)|Microsatellite Instability (MSI):MSS|PD-L1 expression:negative TC<1% IC<1%|Tumor Mutational Burden (TMB):3,97 Muts/MB|ERCC1:Low expression|TYMS:Low expression|	negative| TC<1% IC<1%	3,97	MSS
204	76	Male	ERBB2:c.2524G>A (p.Val842Ile)|SMAD4:c.1082G>A (p.Arg361His)|TP53:c.824G>A (p.Cys275Tyr)|KRAS:amplification|Microsatellite Instability (MSI):MSS|PD-L1 expression:negative TC<1% IC<1%|Tumor Mutational Burden (TMB):6,76 Muts/MB|	negative| TC<1% IC<1%	6,76	MSS

205	51	Male	BRCA2:c.67+1G>A|KRAS:c.35 G>T (p.Gly12Val)|TP53:c.817C>T (p.Arg273Cys)|Microsatellite Instability (MSI):MSS|PD-L1 expression:negative TC<1% IC<1%|Tumor Mutational Burden (TMB): Muts/MB|ERCC1:High expression|TYMS:Low expression|UGT1A1:HOMOZYGOYS MUTATED (7/7)|DPD:WT|	negative| TC<1% IC<1%		MSS
206	63	Male	KRAS:c.35G>A (p.Gly12Asp)|Microsatellite Instability (MSI):MSS|PD-L1 expression:negative TC<1% IC<1%|Tumor Mutational Burden (TMB):4,78 Muts/MB|	negative| TC<1% IC<1%	4,78	MSS
207	49	Female	Microsatellite Instability (MSI):MSS|PD-L1 expression:negative TC<1%, IC<1%|Tumor Mutational Burden (TMB):2,87 Muts/MB|ERCC1:High expression|TYMS:Low expression|UGT1A1:WT (6/6)|DPD:WT|	negative| TC<1%, IC<1%	2,87	MSS

<table>
<tr><td>208</td><td>39</td><td>Female</td><td>KRAS:c.35G>T (p.Gly12Val)|SMAD4:c.403C>T (p.Arg135Ter)|RB1:c.1351_1357delCGCTTGT (p.Arg451IlefsTer4)|TP53:c.985_993+4delACCCTTCAGGTAC (splicing)|Microsatellite Instability (MSI):MSS|PD-L1 expression:negative TC<1% IC<1%|Tumor Mutational Burden (TMB):3,89 Muts/MB|</td><td>negative| TC<1% IC<1%</td><td>3,89</td><td>MSS</td></tr>
<tr><td>209</td><td>42</td><td>Female</td><td>KRAS:c.35G>A (p.Gly12Asp)|TP53:c.159G>A (p.Trp53Ter)|</td><td></td><td></td><td></td></tr>
<tr><td>210</td><td>70</td><td>Male</td><td>KRAS:c.35G>A (p.Gly12Asp)|Microsatellite Instability (MSI):MSS|PD-L1 expression:negative TC<1% IC<1%|Tumor Mutational Burden (TMB):2,86 Muts/MB|</td><td>negative| TC<1% IC<1%</td><td>2,86</td><td>MSS</td></tr>
<tr><td>211</td><td>64</td><td>Female</td><td>KRAS:c.35G>A (p.Gly12Asp)|TP53:c.403T>C (p.Cys135Arg)|Microsatellite Instability (MSI):MSS|PD-L1 expression:negative TC<1%, IC<1%|Tumor Mutational Burden (TMB):2,85 Muts/MB|ERCC1:Low expression|TYMS:Low expression|</td><td>negative| TC<1%, IC<1%</td><td>2,85</td><td>MSS</td></tr>
</table>

<table>
<tr><td>212</td><td>66</td><td>Male</td><td>BAP1:c.125delC (p.Pro42LeufsTer30)|KRAS:c.183A>T (p.Gln61His)|ARID1A:c.2180_2189del (p.Arg727LeufsTer12)|ERCC2:c.1972C>T (p.Arg658Cys)|Microsatellite Instability (MSI):MSS|PD-L1 expression:negative TC<1% IC<1%|Tumor Mutational Burden (TMB):5,3 Muts/MB|ERCC1:High expression|TYMS:Low expression|</td><td>negative|TC<1% IC<1%</td><td>5,3</td><td>MSS</td></tr>
<tr><td>213</td><td>59</td><td>Female</td><td>KRAS:c.35G>A (p.Gly12Asp)|TP53:c.844C>T (p.Arg282Trp)|DPYD:c.1905+1G>A|Microsatellite Instability (MSI):MSS|PD-L1 expression:negative TC<1% IC<1%|Tumor Mutational Burden (TMB):7,58 Muts/MB|</td><td>negative|TC<1% IC<1%</td><td>7,58</td><td>MSS</td></tr>
<tr><td>214</td><td>58</td><td>Female</td><td>KRAS:c.35G>T (p.Gly12Val)|TP53:c.659A>G (p.Tyr220Cys)|EMSY:amplification|Microsatellite Instability (MSI):MSS|PD-L1 expression:positive TC<1% IC 2%|Tumor Mutational Burden (TMB):7,6 Muts/MB|ERCC1:High expression|TYMS:Low expression|</td><td>positive|TC<1% IC 2%</td><td>7,6</td><td>MSS</td></tr>
</table>

215	74	Female	KRAS:c.34G>C (p.Gly12Arg)|TP53:c.646G>A (p.Val216Met)|Microsatellite Instability (MSI):MSS|PD-L1 expression:positive TC<1% IC 10%|Tumor Mutational Burden (TMB):4,77 Muts/MB|ERCC1:Low expression|RRM1:Low expression|UGT1A1:WT (6/6)|DPD:WT|	positive|TC<1% IC 10%	4,77	MSS
216	67	Male	KRAS:c.34G>C (p.Gly12Arg)|SMAD4:c.1081C>T (p.Arg361Cys)|			
217	64	Male	ALK fusion:HIP1(30) - ALK(20)|Microsatellite Instability (MSI):MSS|PD-L1 expression:negative TC<1% IC<1%|Tumor Mutational Burden (TMB):3,79 Muts/MB|	negative| TC<1% IC<1%	3,79	MSS
218	73	Male	KRAS:c.35G>A (p.Gly12Asp)|Microsatellite Instability (MSI):MSS|PD-L1 expression:negative TC<1%, IC<1%|Tumor Mutational Burden (TMB):3,85 Muts/MB|	negative| TC<1%, IC<1%	3,85	MSS
219	57	Male	KRAS:c.35G>T (p.Gly12Val)|TP53:c.510_517 delinsA (p.Glu171Ter)|TP53:c.759_761delCAT (p.Ile255del)|			
220	73	Female	KRAS:c.436G>A (p.Ala146Thr)|			

221	59	Male	Microsatellite Instability (MSI):MSS|PD-L1 expression:negative TC<1%, IC<1%|Tumor Mutational Burden (TMB):3,81 Muts/MB|	negative| TC<1%, IC<1%	3,81	MSS
222	74	Male	KRAS:c.35G>T (p.Gly12Val)|			
223	82	Female	MUTYH:c.1012C>T (p.Gln338Ter)|IDH2:c.514A>T (p.Arg172Trp)|BAP1:c.1123G>T (p.Glu375Ter)|PIK3R1:c.1690A>G (p.Asn564Asp)|TP53:c.374C>A (p.Thr125Lys)|EIF1AX:amplification|SOS1:amplification|STAG2:amplification|Microsatellite Instability (MSI):MSS|PD-L1 expression:negative TC<1%, IC<1%|Tumor Mutational Burden (TMB):9,39 Muts/MB|	negative| TC<1%, IC<1%	9,39	MSS
224	51	Male	Microsatellite Instability (MSI):MSS|Loss of Heterozygosity (LOH):LOHL 0%|PD-L1 expression:negative TC<1% IC<1%|Tumor Mutational Burden (TMB):2,85 Muts/MB|	negative| TC<1% IC<1%	2,85	MSS

225	52	Female	KRAS:c.35G>T (p.Gly12Val)|Microsatellite Instability (MSI):MSS|Loss of Heterozygosity (LOH):LOHH 30,51%|PD-L1 expression:negative TC<1%, IC<1%|Tumor Mutational Burden (TMB):17,96 Muts/MB|ERCC1:Low expression|TYMS:Low expression|	negative| TC<1%, IC<1%	17,96	MSS
226	67	Male	PTEN:c.517C>T (p.Arg173Cys)|Microsatellite Instability (MSI):MSS|PD-L1 expression:negative TC<1%, IC<1%|Tumor Mutational Burden (TMB):3,82 Muts/MB|	negative| TC<1%, IC<1%	3,82	MSS
227	45	Male	KRAS:c.35G>A (p.Gly12Asp)|TP53:c.637C>T (p.Arg213Ter)|Microsatellite Instability (MSI):MSS|PD-L1 expression:negative TC<1%, IC<1%|Tumor Mutational Burden (TMB):2,17 Muts/MB|	negative| TC<1%, IC<1%	2,17	MSS
228	63	Female	KRAS:c.35G>T (p.Gly12Val)|TP53:c.524G>A (p.Arg175His)|Microsatellite Instability (MSI):MSS|PD-L1 expression:- |Tumor Mutational Burden (TMB):6,74 Muts/MB|	-|	6,74	MSS

229	59	Female	CDKN2A:c.233_234delTC (p.Leu78HisfsTer41)|KRAS:c.34G>C (p.Gly12Arg)|Microsatellite Instability (MSI):MSS|Loss of Heterozygosity (LOH):LOHL 0%|PD-L1 expression:negative TC<1%, IC<1%|Tumor Mutational Burden (TMB):0,97 Muts/MB|ERCC1:High expression|TYMS:Low expression|UGT1A1:WT (6/6)|DPD:WT|	negative|TC<1%, IC<1%	0,97	MSS
230	62	Female	KRAS:c.35G>A (p.Gly12Asp)|TP53:c.559+1G>A ()|Microsatellite Instability (MSI):MSS|Loss of Heterozygosity (LOH):LOHL 3,64%|PD-L1 expression:positive TC<1% IC 2%|Tumor Mutational Burden (TMB):4,93 Muts/MB|	positive|TC<1% IC 2%	4,93	MSS

231	49	Female	TP53:c.1001G>T (p.Gly334Val)|BRAF fusion:CCNY(1) - BRAF(10)|Microsatellite Instability (MSI):MSS|Loss of Heterozygosity (LOH):LOHH 28,13%|PD-L1 expression:positive TC<1%, IC 10%|Tumor Mutational Burden (TMB):3,84 Muts/MB|ERCC1:Low expression|TYMS:Low expression|UGT1A1:HOMOZYGOYS MUTATED (7/7)|DPD:WT|	positive|TC<1%, IC 10%	3,84	MSS
232	57	Male	KRAS:c.35G>A (p.Gly12Asp)|TP53:c.587G>C (p.Arg196Pro)|Microsatellite Instability (MSI):MSS|Loss of Heterozygosity (LOH):LOHH 23,21%|PD-L1 expression:positive TC<1% IC 10%|Tumor Mutational Burden (TMB):7,59 Muts/MB|ERCC1:High expression|TYMS:Low expression|	positive|TC<1% IC 10%	7,59	MSS

<table>
<tr><td>233</td><td>70</td><td>Female</td><td>KRAS:c.35G>T (p.Gly12Val)|TP53:c.743G>A (p.Arg248Gln)|PIK3CA:amplification|MECOM:amplification |Microsatellite Instability (MSI):MSS|Loss of Heterozygosity (LOH):LOHL 10,09%|PD-L1 expression:positive TC<1%, IC 2%|Tumor Mutational Burden (TMB):4,79 Muts/MB|</td><td>positive|TC<1%, IC 2%</td><td>4,79</td><td>MSS</td></tr>
<tr><td>234</td><td>73</td><td>Male</td><td>KRAS:c.35G>A (p.Gly12Asp)|Microsatellite Instability (MSI):MSS|PD-L1 expression:negative TC<1%, IC<1%|Tumor Mutational Burden (TMB):4,78 Muts/MB|</td><td>negative| TC<1%, IC<1%</td><td>4,78</td><td>MSS</td></tr>
<tr><td>235</td><td>73</td><td>Male</td><td>KRAS:c.34G>C (p.Gly12Arg)|CCND3:amplification|Microsatellite Instability (MSI):MSS|Loss of Heterozygosity (LOH):LOHH 43,49%|PD-L1 expression:negative TC<1%, IC<1%|Tumor Mutational Burden (TMB):5,75 Muts/MB|</td><td>negative| TC<1%, IC<1%</td><td>5,75</td><td>MSS</td></tr>
<tr><td>236</td><td>56</td><td>Female</td><td>KRAS:c.35G>A (p.Gly12Asp)|Microsatellite Instability (MSI):MSS|PD-L1 expression:negative TC<1% IC<1%|Tumor Mutational Burden (TMB):0,98 Muts/MB|</td><td>negative| TC<1% IC<1%</td><td>0,98</td><td>MSS</td></tr>
</table>

237	70	Male	BLM:c.1720_1735del (p.Ala575ProfsTer38)|KRAS:c.35G>A (p.Gly12Asp)|NOTCH2:amplification|Microsatellite Instability (MSI):MSS|Loss of Heterozygosity (LOH):LOHL 7,63|PD-L1 expression:negative TC<1% IC<1%|Tumor Mutational Burden (TMB):1,94 Muts/MB|	negative| TC<1% IC<1%	1,94	MSS
238	66	Male	KRAS:c.35G>A (p.Gly12Asp)|Microsatellite Instability (MSI):MSS|Loss of Heterozygosity (LOH):LOHL 7,75%|PD-L1 expression:negative TC<1%, IC<1%|Tumor Mutational Burden (TMB):1,9 Muts/MB|	negative| TC<1%, IC<1%	1,9	MSS
239	68	Female	AMER1:c.1138G>T (p.Glu380Ter)|KRAS:c.35G>A (p.Gly12Asp)|TP53:c.602delT (p.Leu201CysfsTer46)|Microsatellite Instability (MSI):MSS|PD-L1 expression:positive TC 20%, IC 15%|Tumor Mutational Burden (TMB):4 Muts/MB|	positive|TC 20%, IC 15%	4	MSS

240	48	Male	KRAS:c.35G>T (p.Gly12Val)|TP53:c.524G>A (p.Arg175His)|Microsatellite Instability (MSI):MSS|PD-L1 expression:negative TC<1% IC<1%|Tumor Mutational Burden (TMB):4,95 Muts/MB|ERCC1:High expression|TYMS:Low expression|UGT1A1:HETEROZYGOUS (6/7)|DPD:WT|	negative| TC<1% IC<1%	4,95	MSS
241	75	Female	KRAS:c.35G>T (p.Gly12Val)|TP53:c.818G>A (p.Arg273His)|Microsatellite Instability (MSI):MSS|PD-L1 expression:negative TC<1% IC<1%|Tumor Mutational Burden (TMB):1,95 Muts/MB|	negative| TC<1% IC<1%	1,95	MSS
242	80	Male	KRAS:c.35G>C (p.Gly12Ala)|Microsatellite Instability (MSI):MSS|PD-L1 expression:negative TC<1% IC<1%|Tumor Mutational Burden (TMB):0,97 Muts/MB|ERCC1:High expression|TYMS:Low expression|	negative| TC<1% IC<1%	0,97	MSS
243	59	Female	KRAS:c.35G>A (p.Gly12Asp)|Microsatellite Instability (MSI):MSS|Loss of Heterozygosity (LOH):LOHL 0%|PD-L1 expression:negative TC<1%, IC<1%|Tumor Mutational Burden (TMB):4,77 Muts/MB|	negative| TC<1%, IC<1%	4,77	MSS

244	41	Male	HRAS:c.351G>T (p.Lys117Asn)|ATRX:c.3200C>A (p.Ser1067Ter)|NOTCH4:c.1845C>A (p.Cys615Ter)|Microsatellite Instability (MSI):MSS|Loss of Heterozygosity (LOH):LOHH 66,58%|PD-L1 expression:negative TC<1%, IC<1%|Tumor Mutational Burden (TMB):12,41 Muts/MB|	negative| TC<1%, IC<1%	12,41	MSS
245	69	Female	KRAS:c.35G>T (p.Gly12Val)|TP53:c.470T>A (p.Val157Asp)|Microsatellite Instability (MSI):MSS|PD-L1 expression:negative TC<1%, IC<1%|Tumor Mutational Burden (TMB):2,85 Muts/MB|	negative| TC<1%, IC<1%	2,85	MSS
246	49	Male	KRAS:c.35G>A (p.Gly12Asp)|Microsatellite Instability (MSI):MSS|PD-L1 expression:positive TC 2% IC 10%|Tumor Mutational Burden (TMB):2.98 Muts/MB|	positive|TC 2% IC 10%	2,98	MSS
247	42	Male	KRAS:c.35G>A (p.Gly12Asp)|Microsatellite Instability (MSI):MSS|PD-L1 expression:negative TC<1% IC<1%|Tumor Mutational Burden (TMB):7,2 Muts/MB|	negative| TC<1% IC<1%	7,2	MSS

248	60	Female	BRCA2:c.7868A>G (p.His2623Arg)|NBN:c.2140C>T (p.Arg714Ter)|SMAD4:c.1140-2A>T (splicing)|Microsatellite Instability (MSI):MSS|Loss of Heterozygosity (LOH):LOHH 34,47|PD-L1 expression:negative TC<1% , IC<1%|Tumor Mutational Burden (TMB): 6.63 Muts/MB|	negative| TC<1% , IC<1%	6,63	MSS
249	77	Female	KRAS:c.35G>A (p.Gly12Asp)|RAD50:c.326_329delCAGA (p.Thr109AsnfsTer20)|ATM:c.5497-2A>G|			
250	56	Female	KRAS:c.34G>C (p.Gly12Arg)|TP53:c.766_767insA (p.Thr256AsnfsTer8)|Microsatellite Instability (MSI):MSS|Loss of Heterozygosity (LOH):LOHL 0%|PD-L1 expression:negative TC<1% IC<1%|Tumor Mutational Burden (TMB):3,83 Muts/MB|	negative| TC<1% IC<1%	3,83	MSS
251	40	Male	KRAS:c.35G>A (p.Gly12Asp)|TP53:c.817C>T (p.Arg273Cys)|Microsatellite Instability (MSI):MSS|Loss of Heterozygosity (LOH):LOHL 0%|PD-L1 expression:negative TC<1% IC<1%|Tumor Mutational Burden (TMB):3,8 Muts/MB|	negative| TC<1% IC<1%	3,8	MSS

252	50	Female	KRAS:c.35G>A (p.Gly12Asp)|TP53:c.701A>G (p.Tyr234Cys)|RB1:c.248delC (p.Ser83LeufsTer28)|PTPRT:c. 1843C>T (p.Gln615Ter)|Microsatellite Instability (MSI):MSS|PD-L1 expression:negative |Tumor Mutational Burden (TMB):1,9 Muts/MB|ERCC1:High expression|TYMS:Low expression|	negative|	1,9	MSS
253	46	Male	KRAS:c.35G>A (p.Gly12Asp)|Microsatellite Instability (MSI):MSS|PD-L1 expression:negative TC<1% IC<1%|Tumor Mutational Burden (TMB):6,65 Muts/MB|ERCC1:High expression|TYMS:Low expression|	negative| TC<1% IC<1%	6,65	MSS
254	68	Male	RAD51C:c.706-2A>G|KRAS:c.35G>A (p.Gly12Asp)|DDR1:c.1039C>T (p.Gln347Ter)|Microsatellite Instability (MSI):MSS|PD-L1 expression:negative TC<1% IC<1%|Tumor Mutational Burden (TMB):3,8 Muts/MB|	negative| TC<1% IC<1%	3,8	MSS

255	81	Male	KRAS:c.35G>A (p.Gly12Asp)|TP53:c.743G>A (p.Arg248Gln)|MGA:c.6984_6987delTGAG (p.Ser2328ArgfsTer6)|CDKN2B fusion:MTAP(5) - CDKN2B-AS1-004(5)|PD-L1 expression:negative TC<1% IC<1%|Tumor Mutational Burden (TMB):3,82 Muts/MB|	negative| TC<1% IC<1%	3,82	
256	72	Female				
257	70	Male	KRAS:c.35G>T (p.Gly12Val)|Microsatellite Instability (MSI):MSS|PD-L1 expression:negative TC<1%, IC<1%|Tumor Mutational Burden (TMB):6,22 Muts/MB|	negative| TC<1%, IC<1%	6,22	MSS
258	71	Male	KRAS:c.35G>T (p.Gly12Val)|TP53:c.310C>T (p.Gln104Ter)|Microsatellite Instability (MSI):MSS|PD-L1 expression:negative TC<1%, IC<1%|Tumor Mutational Burden (TMB):4,49 Muts/MB|	negative| TC<1%, IC<1%	4,49	MSS

259	65	Male	KRAS:c.35G>A (p.Gly12Asp)|UGT1A1:c.211G >A (p.Gly71Arg)|Microsatellite Instability (MSI):MSS|PD-L1 expression:positive IC 15%, TC<1%|Tumor Mutational Burden (TMB):1,27 Muts/MB|ERCC1:High expression|TYMS:Low expression|	positive|IC 15%, TC<1%	1,27	MSS
260	55	Male	KRAS:c.35G>A (p.Gly12Asp)|TP53:c.159_160 insG (p.Phe54ValfsTer3)|			
261	69	Female	KRAS:c.35G>T (p.Gly12Val)|TP53:c.89dupA (p.Asn30LysfsTer13)|Microsatellite Instability (MSI):MSS|Tumor Mutational Burden (TMB):4,77 Muts/MB|		4,77	MSS
262	66	Female	KRAS:c.183A>T (p.Gln61His)|ERBB2:amplification|KRAS:amplification|NTRK3:amplification|Microsatellite Instability (MSI):MSS|Loss of Heterozygosity (LOH):LOHL 12,33%|PD-L1 expression:negative TC<1%, IC<1%|Tumor Mutational Burden (TMB):4,79 Muts/MB|ERCC1:High expression|TYMS:Low expression|UGT1A1:HETEROZYGOUS (6/7)|DPD:WT|	negative| TC<1%, IC<1%	4,79	MSS

263	67	Male	KRAS:c.35G>A (p.Gly12Asp)|TP53:c.713G>T (p.Cys238Phe)|Microsatellite Instability (MSI):MSS|Loss of Heterozygosity (LOH):LOHL 9,41%|PD-L1 expression:positive TC 2%, IC<1%|Tumor Mutational Burden (TMB):1,99 Muts/MB|	positive|TC 2%, IC<1%	1,99	MSS
264	64	Male	KRAS:c.34G>C (p.Gly12Arg)|SMAD4:c.1333C>T (p.Arg445Ter)|CDKN2A:c.196delC (p.His66ThrfsTer80)|TP53:c.112C>T (p.Gln38Ter)|IKBKB:amplification|Microsatellite Instability (MSI):MSS|Loss of Heterozygosity (LOH):LOHL 0%|PD-L1 expression:negative TC<1% IC<1%|Tumor Mutational Burden (TMB):5.71 Muts/MB|	negative|TC<1% IC<1%	5,71	MSS
265	57	Male	KRAS:c.35G>A (p.Gly12Asp)|TP53:c.602_603insT (p.Leu201PhefsTer8)|CCNE1:amplification|AKT2:amplification|AXL:amplification|Microsatellite Instability (MSI):MSS|Loss of Heterozygosity (LOH):LOHL 1.64 %|PD-L1 expression:negative TC <1% IC <1%|Tumor Mutational Burden (TMB):1.9 Muts/MB|	negative|TC <1% IC <1%	1,9	MSS

<table>
<tr><td>266</td><td>63</td><td>Male</td><td>KRAS:c.35G>A (p.Gly12Asp)|BCOR:c.3649C>T (p.Arg1217Ter)|Microsatellite Instability (MSI):MSS|PD-L1 expression:positive TC<1%, IC 5%|Tumor Mutational Burden (TMB):5,83 Muts/MB|</td><td>positive|TC<1%, IC 5%</td><td>5,83</td><td>MSS</td></tr>
<tr><td>267</td><td>62</td><td>Male</td><td>NF1:c.5881A>T (p.Lys1961Ter)|PTPN11:c.205G>A (p.Glu69Lys)|</td><td></td><td></td><td></td></tr>
<tr><td>268</td><td>62</td><td>Female</td><td>KRAS:c.35G>A (p.Gly12Asp)|MSH3:c.2319-1G>A (splicing)|ARID1A:c.4360C>T (p.Gln1454Ter)|Microsatellite Instability (MSI):MSS|PD-L1 expression:negative TC<1% IC<1% |Tumor Mutational Burden (TMB):4.82 Muts/MB|</td><td>negative| TC<1% IC<1%</td><td>4,82</td><td>MSS</td></tr>
<tr><td>269</td><td>78</td><td>Male</td><td>KRAS:c.35G>T (p.Gly12Val)|TP53:c.817C>T (p.Arg273Cys)|Microsatellite Instability (MSI):MSS|PD-L1 expression:positive TC 2%, IC<1%|Tumor Mutational Burden (TMB):2,91 Muts/MB|ERCC1:High expression|TYMS:Low expression|UGT1A1:WT (6/6)|DPD:WT|</td><td>positive|TC 2%, IC<1%</td><td>2,91</td><td>MSS</td></tr>
<tr><td>270</td><td>65</td><td>Male</td><td>KRAS:c.35G>T (p.Gly12Val)|TP53:c.743G>A (p.Arg248Gln)|</td><td></td><td></td><td></td></tr>
</table>

271	76	Female	KRAS:c.35G>A (p.Gly12Asp)|TP53:c.524G>A (p.Arg175His)|Microsatellite Instability (MSI):MSS|PD-L1 expression:negative TC<1% IC<1% |Tumor Mutational Burden (TMB):1.91 Muts/MB|	negative| TC<1% IC<1%	1,91	MSS
272	75	Female	KRAS:c.35G>A (p.Gly12Asp)|TP53:c.920-1G>A (splicing)|			
273	54	Male	KRAS:c.35G>T (p.Gly12Val)|MUTYH:c.89delC (p.Ala30GlufsTer28)|TP53:c.742C>T (p.Arg248Trp)|Microsatellite Instability (MSI):MSS|PD-L1 expression:negative TC<1% IC <1% |Tumor Mutational Burden (TMB): 2.89 Muts/MB|	negative| TC<1% IC <1%	2,89	MSS
274	41	Male	KRAS:c.35G>A (p.Gly12Asp)|SMAD4:c.250-2A>G ()|SMARCA4:c.4620_4627delGAAGATAA (p.Lys1541GlyfsTer37)|Microsatellite Instability (MSI):MSS|Loss of Heterozygosity (LOH):LOHH 25,42%|PD-L1 expression:negative TC<1%, IC<1%|Tumor Mutational Burden (TMB):3,82 Muts/MB|	negative| TC<1%, IC<1%	3,82	MSS
275	79	Male	Microsatellite Instability (MSI):MSS|PD-L1 expression:negative TC<1% IC<1%|Tumor Mutational Burden (TMB):3.14 Muts/MB|	negative| TC<1% IC<1%	3,14	MSS

276	47	Male	KRAS:c.183A>C (p.Gln61His)|TP53:c.844C>T (p.Arg282Trp)|CDH1:c.1565+1 G>A ()|CDH1:c.1320+1G>T ()|KDM6A:c.737delT (p.Leu246TyrfsTer4)|Microsatellite Instability (MSI):MSS|PD-L1 expression:negative TC<1%, IC<1%|Tumor Mutational Burden (TMB):3,18 Muts/MB|	negative| TC<1%, IC<1%	3,18	MSS
277	59	Female	KRAS:c.34G>C (p.Gly12Arg)|ARID1A:c.31_56 del (p.Ser11AlafsTer91)|KDR:c.10 62C>A (p.Tyr354Ter)|TP53:c.743G>A (p.Arg248Gln)|FANCG:amplification|Microsatellite Instability (MSI):MSS|PD-L1 expression:negative TC <1% IC<1%|Tumor Mutational Burden (TMB):2.92 Muts/MB|ERCC1:High expression|TYMS:Low expression|UGT1A1:HETEROZYGOUS (6/7)|DPD:WT|	negative| TC <1% IC<1%	2,92	MSS
278	60	Male	KRAS:c.183A>C (p.Gln61His)|Microsatellite Instability (MSI):MSS|PD-L1 expression:negative TC<1%, IC<1%|Tumor Mutational Burden (TMB):7,02 Muts/MB|	negative| TC<1%, IC<1%	7,02	MSS

279	72	Female	KRAS:c.34G>C (p.Gly12Arg)|Microsatellite Instability (MSI):MSS|Loss of Heterozygosity (LOH):LOHL 0%|PD-L1 expression:negative TC<1%, IC<1%|Tumor Mutational Burden (TMB):0,95 Muts/MB|	negative| TC<1%, IC<1%	0,95	MSS
280	66	Female	KRAS:c.35G>C (p.Gly12Ala)|TP53:c.742C>T (p.Arg248Trp)|Microsatellite Instability (MSI):MSS|Loss of Heterozygosity (LOH):LOHH 36.49 %|PD-L1 expression:positive TC<1% IC 5%|Tumor Mutational Burden (TMB):1.89 Muts/MB|	positive|TC<1% IC 5%	1,89	MSS
281	61	Female	KRAS:c.34G>C (p.Gly12Arg)|SMAD4:c.362T>A (p.Leu121Ter)|TP53:c.817C>T (p.Arg273Cys)|STAG2:c.1229G>C (p.Cys410Ser)|ABRAXAS1:c.1133_1134insCCCAGTAA (p.Gln379ProfsTer10)|Microsatellite Instability (MSI):MSS|Loss of Heterozygosity (LOH):LOHH 27,76|PD-L1 expression:negative TC<1%, IC<1%|Tumor Mutational Burden (TMB):10,44 Muts/MB|	negative| TC<1%, IC<1%	10,44	MSS

282	84	Female	KRAS:c.35G>A (p.Gly12Asp)|DPYD:c.1905+1 G>A|TP53:c.817C>T (p.Arg273Cys)|Microsatellite Instability (MSI):MSS|Loss of Heterozygosity (LOH):LOHL 15,76%|PD-L1 expression:negative TC<1%, IC<1%|Tumor Mutational Burden (TMB):3,79 Muts/MB|	negative| TC<1%, IC<1%	3,79	MSS
283	62	Male	MRE11A:c.1252_1253insA (p.Ile418AsnfsTer39)|KRAS:c.35G>T (p.Gly12Val)|ID3:amplification|MCL1:amplification|ELF3:amplification|H3-3A:amplification|PCBP1:amplification|H3C2:amplification|H1-4:amplification|PIM1:amplification|MET:amplification|FANCF:amplification|CDKN1B:amplification|KNSTRN:amplification|GNA13:amplification|H3-3B:amplification|Microsatellite Instability (MSI):MSS|Loss of Heterozygosity (LOH):LOHH 42.67 %|PD-L1 expression:negative TC<1% IC<1%|Tumor Mutational Burden (TMB):2.85 Muts/MB|	negative| TC<1% IC<1%	2,85	MSS

284	64	Male	KRAS:c.35G>A (p.Gly12Asp)|TP53:c.524G>A (p.Arg175His)|Microsatellite Instability (MSI):MSS|Loss of Heterozygosity (LOH):LOHL 2.42 %|PD-L1 expression:negative TC <1% IC<1% |Tumor Mutational Burden (TMB):0.95 Muts/MB|	negative| TC <1% IC<1%	0,95	MSS
285	57	Female	KRAS:c.35G>A (p.Gly12Asp)|Microsatellite Instability (MSI):MSS|PD-L1 expression:positive TC<1%, IC 1%|Tumor Mutational Burden (TMB):1,89 Muts/MB|ERCC1:Low expression|TYMS:Low expression|UGT1A1:HOMOZYGOYS MUTATED (7/7)|DPD:WT|	positive|TC<1%, IC 1%	1,89	MSS
286	58	Male	KRAS:c.34G>C (p.Gly12Arg)|TP53:c.824G>A (p.Cys275Tyr)|Microsatellite Instability (MSI):MSS|Loss of Heterozygosity (LOH):LOHL 7,61%|PD-L1 expression:negative TC<1%, IC<1%|Tumor Mutational Burden (TMB):2,85 Muts/MB|ERCC1:Low expression|TYMS:Low expression|UGT1A1:HETEROZYGOUS (6/7)|DPD:WT|	negative| TC<1%, IC<1%	2,85	MSS

287	58	Female	KRAS:c.35G>A (p.Gly12Asp)|RAD50:c.326_329delCAGA (p.Thr109AsnfsTer20)|			
288	52	Female	KRAS:c.35G>A (p.Gly12Asp)|Microsatellite Instability (MSI):MSS|Loss of Heterozygosity (LOH):LOHL 0%|PD-L1 expression:negative TC<1%, IC<1%|Tumor Mutational Burden (TMB):6,62 Muts/MB|ERCC1:Low expression|TYMS:Low expression|UGT1A1:HETEROZYGOUS (6/7)|DPD:WT|	negative| TC<1%, IC<1%	6,62	MSS
289	50	Male	KRAS:c.35G>T (p.Gly12Val)|TP53:c.808T>C (p.Phe270Leu)|FGFR3 fusion:FGFR3-TACC3|Microsatellite Instability (MSI):MSS|Loss of Heterozygosity (LOH):LOHL 8,65%|PD-L1 expression:positive TC 30%, IC 5%|Tumor Mutational Burden (TMB):2,85 Muts/MB|	positive|TC 30%, IC 5%	2,85	MSS
290	79	Male	PIK3CA:c.1633G>A (p.Glu545Lys)|			

291	58	Female	KRAS:c.35G>T (p.Gly12Val)|RNF43:c.1204C>T (p.Gln402Ter)|TP53:c.455C>T (p.Pro152Leu)|Microsatellite Instability (MSI):MSS|Loss of Heterozygosity (LOH):LOHL 5.33 %|PD-L1 expression:negative TC <1% IC<1% |Tumor Mutational Burden (TMB):1.91 Muts/MB|	negative| TC <1% IC<1%	1,91	MSS
292	56	Male	NBN:c.654_658delAAAAC (p.Lys219AsnfsTer16)|FGFR1:c.1731C>G (p.Asn577Lys)|TERT:amplification|Microsatellite Instability (MSI):MSS|Loss of Heterozygosity (LOH):LOHH 62,57%|PD-L1 expression:negative TC<1%, IC<1%|Tumor Mutational Burden (TMB):3,8 Muts/MB|	negative| TC<1%, IC<1%	3,8	MSS
293	65	Male	KRAS:c.35G>T (p.Gly12Val)|TP53:c.380C>T (p.Ser127Phe)|Microsatellite Instability (MSI):MSS|Loss of Heterozygosity (LOH):LOHL 13.64 %|PD-L1 expression:negative TC <1% IC<1% |Tumor Mutational Burden (TMB):2.85 Muts/MB|	negative| TC <1% IC<1%	2,85	MSS

294	59	Female	KRAS:c.34G>C (p.Gly12Arg)|Microsatellite Instability (MSI):MSS|Tumor Mutational Burden (TMB):0,95 Muts/MB|		0,95	MSS
295	57	Male	KRAS:c.35G>A (p.Gly12Asp)|SMAD4:c.1096C >T (p.Gln366Ter)|Microsatellite Instability (MSI):MSS|Loss of Heterozygosity (LOH):LOHH 26,46%|PD-L1 expression:negative TC<1%, IC<1%|Tumor Mutational Burden (TMB):4,74 Muts/MB|ERCC1:High expression|TYMS:Low expression|UGT1A1:WT (6/6)|DPD:WT|	negative| TC<1%, IC<1%	4,74	MSS
296	54	Male	KRAS:c.34G>T (p.Gly12Cys)| GNAS:c.602G>A (p.Arg201His)|ARID1A:c.1780 C>T (p.Gln594Ter)|NOTCH4:c.104 4delC (p.Gly349AlafsTer49)|Microsa tellite Instability (MSI):MSS|Tumor Mutational Burden (TMB):4.76 Muts/MB|		4,76	MSS

297	57	Female	KRAS:c.183A>C (p.Gln61His)|TP53:c.378C>G (p.Tyr126Ter)|H3C2:amplification|Microsatellite Instability (MSI):MSS|Loss of Heterozygosity (LOH):LOHL 0%|PD-L1 expression:negative TC<1%, IC<1%|Tumor Mutational Burden (TMB):4,73 Muts/MB|ERCC1:Low expression|TYMS:Low expression|UGT1A1:WT (6/6)|DPD:WT|	negative|TC<1%, IC<1%	4,73	MSS
298	59	Male	EPHA2:c.1969C>T (p.Arg657Ter)|PIK3CA:c.3140A>G (p.His1047Arg)|MYC:amplification|Microsatellite Instability (MSI):MSS|PD-L1 expression:positive TC 2%, IC<1%|Tumor Mutational Burden (TMB):5,69 Muts/MB|	positive|TC 2%, IC<1%	5,69	MSS
299	74	Male	BARD1:c.947T>G (p.Leu316Ter)|AMER1:c.1489C>T (p.Arg497Ter)|Microsatellite Instability (MSI):MSS|PD-L1 expression:negative TC<1% IC<1%|Tumor Mutational Burden (TMB): Muts/MB|ERCC1:High expression|TYMS:Low expression|UGT1A1:HETEROZYGOUS (6/7)|DPD:WT|	negative|TC<1% IC<1%		MSS

<u>300</u>	49	Female	KRAS:c.35G>A (p.Gly12Asp)|TP53:c.524G>A (p.Arg175His)|SMAD4:c.133A >G (p.Lys45Glu)|TGFBR2:c.1232T >C (p.Leu411Pro)|Microsatellite Instability (MSI):MSS|Loss of Heterozygosity (LOH):LOHL 2,19%|PD-L1 expression:negative TC<1%, IC<1%|Tumor Mutational Burden (TMB):4,74 Muts/MB|	negative| TC<1%, IC<1%	4,74	MSS
<u>301</u>	67	Female	TP53:c.158G>A (p.Trp53Ter)|Microsatellite Instability (MSI):MSS|Loss of Heterozygosity (LOH):LOHH 16,16%|PD-L1 expression:negative TC<1%, IC<1%|Tumor Mutational Burden (TMB):4,74 Muts/MB|ERCC1:Low expression|TYMS:Low expression|	negative| TC<1%, IC<1%	4,74	MSS
<u>302</u>	70	Female	KRAS:c.35G>T (p.Gly12Val)|TP53:c.659A>G (p.Tyr220Cys)|NCOR1:c.1519 C>T (p.Arg507Ter)|Microsatellite Instability (MSI):MSS|PD-L1 expression:positive TC<1%, IC 3%|Tumor Mutational Burden (TMB):2,84 Muts/MB|	positive|T C<1%, IC 3%	2,84	MSS

303	64	Female	KRAS:c.34G>C (p.Gly12Arg)|TP53:c.404G>A (p.Cys135Tyr)|Microsatellite Instability (MSI):MSS|PD-L1 expression:negative TC 1% IC <1% |Tumor Mutational Burden (TMB):3.79 Muts/MB|ERCC1:High expression|TYMS:Low expression|	negative| TC 1% IC <1%	3,79	MSS
304	59	Male	KRAS:c.35G>A (p.Gly12Asp)|TP53:c.961A>T (p.Lys321Ter)|SF3B1:c.2098A >G (p.Lys700Glu)|AURKC:c.420de lA (p.Glu140AspfsTer7)|MYC:am plification|Microsatellite Instability (MSI):MSS|Loss of Heterozygosity (LOH):LOHH 32,66%|PD-L1 expression:positive TC 2%, IC 1%|Tumor Mutational Burden (TMB):2,85 Muts/MB|ERCC1:High expression|TYMS:Low expression|	positive|T C 2%, IC 1%	2,85	MSS
305	60	Female	KRAS:c.35G>A (p.Gly12Asp)|TP53:c.743G>A (p.Arg248Gln)|			

306	78	Male	KRAS:c.182A>G (p.Gln61Arg)|SF3B1:c.2098A>G (p.Lys700Glu)|TP53:c.844C>T (p.Arg282Trp)|BCL2L12:-|Microsatellite Instability (MSI):MSS|Loss of Heterozygosity (LOH):LOHH 38.87 %|PD-L1 expression:positive TC 20% IC 5%|Tumor Mutational Burden (TMB):3.79 Muts/MB|	positive|TC 20% IC 5%	3,79	MSS
307	76	Female	KRAS:c.35G>A (p.Gly12Asp)|CDC73:c.131+1G>T (splicing)|RBM10:c.2661delA (p.Glu888LysfsTer24)|CDKN1A:c.206_207insCG (p.Leu71AlafsTer78)|Microsatellite Instability (MSI):MSS|Loss of Heterozygosity (LOH):LOHL 0%|PD-L1 expression:positive TC<1%, IC 5%|Tumor Mutational Burden (TMB):5,71 Muts/MB|	positive|TC<1%, IC 5%	5,71	MSS
308	66	Female	ARID1B:c.1618C>T (p.Gln540Ter)|Microsatellite Instability (MSI):MSS|Tumor Mutational Burden (TMB):fail Muts/MB|		fail	MSS

309	74	Female	KRAS:c.35G>T (p.Gly12Val)|TP53:c.711G>T (p.Met237Ile)|Microsatellite Instability (MSI):MSS|Loss of Heterozygosity (LOH):LOHH 37,87%|PD-L1 expression:negative TC<1%, IC<1%|Tumor Mutational Burden (TMB):0,95 Muts/MB|	negative| TC<1%, IC<1%	0,95	MSS
310	52	Female	KRAS:c.35G>T (p.Gly12Val)|CDKN2A:c.150+1 G>T (splicing)|Microsatellite Instability (MSI):MSS|Loss of Heterozygosity (LOH):LOHL 9.58 %|PD-L1 expression:negative TC <1% IC<1%|Tumor Mutational Burden (TMB):2.84 Muts/MB|	negative| TC <1% IC<1%	2,84	MSS
311	77	Female	KRAS:c.35G>A (p.Gly12Asp)|TP53:c.722C>G (p.Ser241Cys)|SMAD4:c.1082 G>A (p.Arg361His)|CDKN2A:c.311 delT (p.Leu104ArgfsTer42)|Microsatellite Instability (MSI):MSS|Loss of Heterozygosity (LOH):LOHL 0%|PD-L1 expression:positive TC 5%, IC<1%|Tumor Mutational Burden (TMB):4,75 Muts/MB|	positive|TC 5%, IC<1%	4,75	MSS

312	37	Female	KRAS:c.35G>T (p.Gly12Val)|Microsatellite Instability (MSI):MSS|PD-L1 expression:negative TC<1% IC<1%|Tumor Mutational Burden (TMB):11.7 Muts/MB|ERCC1:High expression|TYMS:Low expression|UGT1A1:HETEROZYGOUS (6/7)|DPD:WT|	negative| TC<1% IC<1%	11,7	MSS
313	73	Male	KRAS:c.35G>A (p.Gly12Asp)|FBXW7:c.1436G>A (p.Arg479Gln)|CREBBP:c.4232G>T (p.Gly1411Val)|Microsatellite Instability (MSI):MSS|PD-L1 expression:negative TC<1%, IC<1%|Tumor Mutational Burden (TMB):6,65 Muts/MB|	negative| TC<1%, IC<1%	6,65	MSS
314	37	Male	Microsatellite Instability (MSI):MSS|Loss of Heterozygosity (LOH):LOHH 19.62 %|PD-L1 expression:negative TC <1%IC<1%|Tumor Mutational Burden (TMB):1.89 Muts/MB|	negative| TC <1%IC<1%	1,89	MSS
315	53	Female	KRAS:c.35G>A (p.Gly12Asp)|TP53:c.374C>A (p.Thr125Lys)|Microsatellite Instability (MSI):MSS|Tumor Mutational Burden (TMB):4.74 Muts/MB|		4,74	MSS

316	74	Male	KRAS:c.35G>T (p.Gly12Val)|TP53:c.342_343insG (p.His115AlafsTer34)|Microsatellite Instability (MSI):MSS|Loss of Heterozygosity (LOH):LOHL 1.59 %|PD-L1 expression:positive TC 25% IC <1%|Tumor Mutational Burden (TMB):5.69 Muts/MB|	positive|TC 25% IC <1%	5,69	MSS
317	57	Male				
318	54	Male	KRAS:c.34G>C (p.Gly12Arg)|TP53:c.592G>T (p.Glu198Ter)|SMAD4:c.1051G>T (p.Asp351Tyr)|Microsatellite Instability (MSI):MSS|PD-L1 expression negative TC<1%, IC<1%|Tumor Mutational Burden (TMB):1,9 Muts/MB|	positive|TC<1%, IC<1%	1,9	MSS
319	61	Female	KRAS:c.34G>C (p.Gly12Arg)|TP53:c.844C>T (p.Arg282Trp)|Microsatellite Instability (MSI):MSS|Loss of Heterozygosity (LOH):LOHL 0%|PD-L1 expression:negative TC<1%, IC<1%|Tumor Mutational Burden (TMB):1,9 Muts/MB|ERCC1:Low expression|TYMS:Low expression|UGT1A1:WT (6/6)|DPD:WT|	negative|TC<1%, IC<1%	1,9	MSS

320	58	Female	KRAS:c.35G>A (p.Gly12Asp)|Microsatellite Instability (MSI):MSS|Loss of Heterozygosity (LOH):LOHL 5,52%|PD-L1 expression:positive TC 10%, IC 1%|Tumor Mutational Burden (TMB):5,7 Muts/MB|	positive|TC 10%, IC 1%	5,7	MSS
321	79	Male	KRAS:c.35G>A (p.Gly12Asp)|TP53:c.587G>C (p.Arg196Pro)|Microsatellite Instability (MSI):MSS|Loss of Heterozygosity (LOH):LOHL 2,39%|PD-L1 expression:positive TC 10%, IC<1%|Tumor Mutational Burden (TMB):3,81 Muts/MB|	positive|TC 10%, IC<1%	3,81	MSS
322	69	Female	KRAS:c.35G>T (p.Gly12Val)|SMAD4:c.1066C>A (p.Pro356Thr)|TGFBR2:c.1115T>C (p.Leu372Pro)|Microsatellite Instability (MSI):MSS|Loss of Heterozygosity (LOH):LOHL 0,79%|PD-L1 expression:negative TC<1%, IC<1%|Tumor Mutational Burden (TMB):3,8 Muts/MB|	negative|TC<1%, IC<1%	3,8	MSS
323	52	Male	Microsatellite Instability (MSI):MSS|PD-L1 expression:negative TC<1%, IC<1%|Tumor Mutational Burden (TMB):0,95 Muts/MB|	negative|TC<1%, IC<1%	0,95	MSS

324	49	Female	IDH1:c.395G>T (p.Arg132Leu)|MAP2K1:c.158 T>G (p.Phe53Cys)|EPHA2:c.594C> G (p.Tyr198Ter)|Microsatellite Instability (MSI):MSS|Loss of Heterozygosity (LOH):LOHH 24,18%|PD-L1 expression:negative TC<1%, IC<1%|Tumor Mutational Burden (TMB):3,8 Muts/MB|	negative| TC<1%, IC<1%	3,8	MSS
325	70	Male	KRAS:c.35G>A (p.Gly12Asp)|MUTYH:c.734G> A (p.Arg245His)|TP53:c.925_94 1del (p.Pro309LeufsTer22)|Micros atellite Instability (MSI):MSS|Loss of Heterozygosity (LOH):LOHH 32,61%|PD-L1 expression:negative TC<1%, IC<1%|Tumor Mutational Burden (TMB):0,95 Muts/MB|	negative| TC<1%, IC<1%	0,95	MSS
326	71	Female	KRAS:c.34G>C (p.Gly12Arg)|Microsatellite Instability (MSI):MSS|PD-L1 expression:negative TC <1% IC<1%|Tumor Mutational Burden (TMB):3.83 Muts/MB|	negative| TC <1% IC<1%	3,83	MSS

327	72	Male	KRAS:c.35G>T (p.Gly12Val)|Microsatellite Instability (MSI):MSS|Loss of Heterozygosity (LOH):LOHL 0%|PD-L1 expression:positive TC<1% IC 2%|Tumor Mutational Burden (TMB):0 Muts/MB|	positive|TC<1% IC 2%	0	MSS
328	48	Female	RAD51:c.817C>T (p.Gln273Ter)|KRAS:c.35G>A (p.Gly12Asp)|TP53:c.824_833 del (p.Cys275LeufsTer67)|Microsatellite Instability (MSI):MSS|Loss of Heterozygosity (LOH):LOHL 11,06%|PD-L1 expression:negative TC<1%, IC<1%|Tumor Mutational Burden (TMB):6,64 Muts/MB|	negative| TC<1%, IC<1%	6,64	MSS
329	67	Female	KRAS:c.35G>A (p.Gly12Asp)|RECQL4:c.3236+1G>C (splicing)|U2AF1:c.101C>T (p.Ser34Phe)|TP53:c.374C>T (p.Thr125Met)|Microsatellite Instability (MSI):MSS|PD-L1 expression:negative TC<1% IC<1%|Tumor Mutational Burden (TMB):5.68 Muts/MB|	negative| TC<1% IC<1%	5,68	MSS

330	66	Male	KRAS:c.34G>C (p.Gly12Arg)|TP53:c.818G>T (p.Arg273Leu)|Microsatellite Instability (MSI):MSS|PD-L1 expression:negative TC<1% IC<1%|Tumor Mutational Burden (TMB):1.9 Muts/MB|	negative| TC<1% IC<1%	1,9	MSS
331	74	Male	KRAS:c.35G>A (p.Gly12Asp)|TP53:c.423_424 delCCinsG (p.Cys141TrpfsTer29)|Microsatellite Instability (MSI):MSS|Loss of Heterozygosity (LOH):LOHL 1.24 %|PD-L1 expression:positive TC 1% IC<1%|Tumor Mutational Burden (TMB):2.85 Muts/MB|	positive|TC 1% IC<1%	2,85	MSS
332	47	Female	Microsatellite Instability (MSI):MSS|PD-L1 expression:negative TC <1% IC<1%|Tumor Mutational Burden (TMB):3.78 Muts/MB|	negative| TC <1% IC<1%	3,78	MSS
333	53	Female	KRAS:c.35G>T (p.Gly12Val)|MPL:amplification|TP63:amplification|AR:amplification|Microsatellite Instability (MSI):MSS|Loss of Heterozygosity (LOH):LOHL 5.74 %|PD-L1 expression:negative TC <1% IC<1%|Tumor Mutational Burden (TMB):4.76 Muts/MB|	negative| TC <1% IC<1%	4,76	MSS

334	51	Male	KRAS:c.35G>T (p.Gly12Val)|SMAD4:c.1081C >T (p.Arg361Cys)|FAT1:c.4587_4 588insCT (p.Thr1531SerfsTer12)|PARP2 :amplification|Microsatellite Instability (MSI):MSS|Loss of Heterozygosity (LOH):LOHH 20.65 %|PD-L1 expression:negative TC<1% IC<1%|Tumor Mutational Burden (TMB):7.57 Muts/MB|	negative| TC<1% IC<1%	7,57	MSS
335	70	Male	KRAS:c.35G>A (p.Gly12Asp)|ARID1A:c.5573_ 5576delACTT (p.His1858ProfsTer24)|TP53:c .707A>G (p.Tyr236Cys)|Microsatellite Instability (MSI):MSS|Loss of Heterozygosity (LOH):LOHL 9.06 %|PD-L1 expression:negative TC <1% IC<1%|Tumor Mutational Burden (TMB):2.84 Muts/MB|	negative| TC <1% IC<1%	2,84	MSS
336	53	Male	KRAS:c.35G>A (p.Gly12Asp)|TP53:c.42delG (p.Ser15ValfsTer29)|Microsat ellite Instability (MSI):MSS|Loss of Heterozygosity (LOH):LOHL 6.69 %|PD-L1 expression:negative TC <1% IC<1%|Tumor Mutational Burden (TMB):4.73 Muts/MB|	negative| TC <1% IC<1%	4,73	MSS

337	65	Male	KRAS:c.35G>A (p.Gly12Asp)|TP53:c.428T>C (p.Val143Ala)|TGFBR2:c.1558 C>T (p.Arg520Ter)|RASA2:c.1359+ 2T>C (splicing)|Microsatellite Instability (MSI):MSS|Loss of Heterozygosity (LOH):LOHL 11.11 %|PD-L1 expression:- |Tumor Mutational Burden (TMB):4.73 Muts/MB|ERCC1:Low expression|TYMS:Low expression|UGT1A1:HETEROZ YGOUS (6/7)|DPD:WT|	-|	4,73	MSS
338	68	Male	NRAS:c.182A>G (p.Gln61Arg)|BAP1:c.287_288 delTG (p.Leu96SerfsTer29)|TP53:c.4 9delG (p.Glu17LysfsTer27)|Microsat ellite Instability (MSI):MSS|Loss of Heterozygosity (LOH):LOHL 2.57 %|PD-L1 expression:negative TC <1% IC<1%|Tumor Mutational Burden (TMB):3.79 Muts/MB|	negative| TC <1% IC<1%	3,79	MSS

339	71	Male	KRAS:c.34G>C (p.Gly12Arg)|ASXL1:c.427C>T (p.Arg143Ter)|KRAS:amplification|Microsatellite Instability (MSI):MSS|Loss of Heterozygosity (LOH):LOHH 27,82%|PD-L1 expression:positive TC 30%, IC 15%|Tumor Mutational Burden (TMB):7,59 Muts/MB|	positive|TC 30%, IC 15%	7,59	MSS
340	51	Female	KRAS:c.35G>T (p.Gly12Val)|RB1:c.1547G>A (p.Trp516Ter)|			
341	73	Male	KRAS:c.35G>A (p.Gly12Asp)|CDKN2A:c.151-2A>G (splicing)|TGFBR2:c.190_191insA (p.Thr64AsnfsTer2)|Microsatellite Instability (MSI):MSS|Loss of Heterozygosity (LOH):LOHL 0 %|PD-L1 expression:negative TC<1% IC<1%|Tumor Mutational Burden (TMB):0.95 Muts/MB|	negative| TC<1% IC<1%	0,95	MSS
342	65	Male	KRAS:c.35G>A (p.Gly12Asp)|Microsatellite Instability (MSI):MSS|Loss of Heterozygosity (LOH):LOHH 33,06%|PD-L1 expression:negative TC<1%, IC<1%|Tumor Mutational Burden (TMB):3,79 Muts/MB|ERCC1:High expression|TYMS:Low expression|	negative| TC<1%, IC<1%	3,79	MSS

343	63	Female	KRAS:c.34G>C (p.Gly12Arg)|Microsatellite Instability (MSI):-|Genomic Instability Score (GIS):GISL 3|Tumor Mutational Burden (TMB):1,89 Muts/MB|		1,89	-
344	59	Female	MRE11A:c.1252_1253insA (p.Ile418AsnfsTer39)|KRAS:c.34G>C (p.Gly12Arg)|DPYD:c.1905+1G>A|TP53:c.192delC (p.Arg65GlufsTer58)|Microsatellite Instability (MSI):MSS|Loss of Heterozygosity (LOH):LOHH 23,95%|PD-L1 expression:positive TC<1%, IC 1%|Tumor Mutational Burden (TMB):0,95 Muts/MB|ERCC1:High expression|TYMS:Low expression|UGT1A1:HOMOZYGOYS MUTATED (7/7)|DPD:HETEROZYGOUS|	positive|TC<1%, IC 1%	0,95	MSS
345	53	Male	KRAS:c.35G>A (p.Gly12Asp)|Microsatellite Instability (MSI):MSS|Loss of Heterozygosity (LOH):LOHL 0%|PD-L1 expression:negative TC<1%, IC<1%|Tumor Mutational Burden (TMB):8,55 Muts/MB|	negative|TC<1%, IC<1%	8,55	MSS

346	58	Male	KRAS:c.38G>A (p.Gly13Asp)|PMS1:c.793C>T (p.Arg265Ter)|TP53:c.488A>G (p.Tyr163Cys)|Microsatellite Instability (MSI):MSS|Loss of Heterozygosity (LOH):LOHL 0 %|PD-L1 expression:positive TC <1% IC 15%|Tumor Mutational Burden (TMB):9.51 Muts/MB|	positive|TC <1% IC 15%	9,51	MSS
347	51	Male	FANCL:c.2T>C (p.Met1?)|FGFR2 fusion:FGFR2(17) - CCDC6(2)|Microsatellite Instability (MSI):MSS|Loss of Heterozygosity (LOH):LOHH 38,17%|PD-L1 expression:negative TC<1%, IC<1%|Tumor Mutational Burden (TMB):2,84 Muts/MB|	negative| TC<1%, IC<1%	2,84	MSS
348	66	Female	KRAS:c.34G>T (p.Gly12Cys)|SMAD4:c.1309-3A>G ()|RNF43:c.153_154insATCA (p.Pro52IlefsTer24)|RNASEH2B:c.3G>A (p.Met1?)|Microsatellite Instability (MSI):MSS|Loss of Heterozygosity (LOH):LOHH 16.61 %|PD-L1 expression:negative TC <1% IC<1%|Tumor Mutational Burden (TMB):6.65 Muts/MB|	negative| TC <1% IC<1%	6,65	MSS

349	71	Female	KRAS:c.35G>T (p.Gly12Val)|DPYD:c.1905+1G>A|BLM:c.1882+1G>A|KMT2C:c.11460+1delG (splicing)|TP53:c.473_473delGinsTT (p.Arg158LeufsTer23)|Microsatellite Instability (MSI):MSS|Loss of Heterozygosity (LOH):LOHL 10.74 %|PD-L1 expression:negative TC <1% IC<1%|Tumor Mutational Burden (TMB):2.86 Muts/MB|	negative| TC <1% IC<1%	2,86	MSS
350	66	Male	KRAS:c.34G>C (p.Gly12Arg)|			
351	55	Female	KRAS:c.35G>A (p.Gly12Asp)|SMAD4:c.1096C>T (p.Gln366Ter)|TP53:c.1013del (p.Phe338SerfsTer7)|Microsatellite Instability (MSI):MSS|Loss of Heterozygosity (LOH):LOHL 10.02 %|PD-L1 expression:negative TC<1% IC<1%|Tumor Mutational Burden (TMB):2.84 Muts/MB|ERCC1:Low expression|TYMS:Low expression|	negative| TC<1% IC<1%	2,84	MSS

352	61	Male	PALB2:c.719delC (p.Pro240LeufsTer39)|KRAS:c.35G>T (p.Gly12Val)|SMAD4:c.533C>G (p.Ser178Ter)|PARP1:c.2848+3A>G ()|Microsatellite Instability (MSI):MSS|Loss of Heterozygosity (LOH):LOHH 33.95 %|PD-L1 expression:negative TC <1% IC<1%|Tumor Mutational Burden (TMB):7.59 Muts/MB|ERCC1:High expression|TYMS:Low expression|	negative| TC <1% IC<1%	7,59	MSS
353	48	Male	PTEN:c.760A>T (p.Lys254Ter)|APC:c.4435delG (p.Val1479SerfsTer28)|Microsatellite Instability (MSI):MSS|PD-L1 expression:negative TC <1% IC<1%|Tumor Mutational Burden (TMB):FAIL Muts/MB|	negative| TC <1% IC<1%	FAIL	MSS
354	68	Female	KRAS:c.35G>A (p.Gly12Asp)|TP53:c.777_778insC (p.Ser260LeufsTer4)|Microsatellite Instability (MSI):MSS|Loss of Heterozygosity (LOH):LOHL 0 %|PD-L1 expression:negative TC <1% IC<1%|Tumor Mutational Burden (TMB):3.8 Muts/MB|	negative| TC <1% IC<1%	3,8	MSS

355	62	Male	BRCA2:BRCA2 gene duplication|KRAS:c.35G>A (p.Gly12Asp)|SMAD4:c.1336C>T (p.Gln446Ter)|JAK2:amplification|Microsatellite Instability (MSI):MSS|Loss of Heterozygosity (LOH):LOHH 31%|PD-L1 expression:negative TC<1% IC<1%|Tumor Mutational Burden (TMB):5.7 Muts/MB|	negative| TC<1% IC<1%	5,7	MSS
356	63	Female	KRAS:c.34G>T (p.Gly12Cys)|DPYD:c.1905+1G>A|STK11:c.527A>T (p.Asp176Val)|STK11:c.527A>T (p.Asp176Val)|TP53:c.853G>A (p.Glu285Lys)|Microsatellite Instability (MSI):MSS|Loss of Heterozygosity (LOH):LOHL 0 %|PD-L1 expression:positive TC 1% IC<1%|Tumor Mutational Burden (TMB):9.49 Muts/MB|ERCC1:High expression|TYMS:Low expression|UGT1A1:HETEROZYGOUS (6/7)|DPD:HETEROZYGOUS|	positive|TC 1% IC<1%	9,49	MSS
357	83	Female	KRAS:c.34G>C (p.Gly12Arg)|TP53:c.499C>T (p.Gln167Ter)|			

358	47	Female	KRAS:c.35G>T (p.Gly12Val)|CHEK2:c.470T>C (p.Ile157Thr)|CDKN2A:c.291_292delGC (p.His98ProfsTer21)|TP53:c.844C>T (p.Arg282Trp)|TP53:c.578A>T (p.His193Leu)|Microsatellite Instability (MSI):MSS|PD-L1 expression:negative TC<1% IC<1%|Tumor Mutational Burden (TMB):8.65 Muts/MB|	negative| TC<1% IC<1%	8,65	MSS
359	68	Male	BRCA2:|BRAF:c.1462_1476delACAGCACCTACACCT (p.Thr488_Pro492del)|TP53:c.659A>G (p.Tyr220Cys)|CCNE1:amplification|Microsatellite Instability (MSI):MSS|Loss of Heterozygosity (LOH):LOHH 34.81 %|PD-L1 expression:negative TC <1% IC<1%|Tumor Mutational Burden (TMB):0.95 Muts/MB|	negative| TC <1% IC<1%	0,95	MSS

360	54	Female	KRAS:c.35G>A (p.Gly12Asp)|SMAD4:c.1333C>T (p.Arg445Ter)|TP53:c.725G>A (p.Cys242Tyr)|Microsatellite Instability (MSI):MSS|PD-L1 expression:positive TC 5%, IC<1%|Tumor Mutational Burden (TMB):1,9 Muts/MB|ERCC1:Low expression|TYMS:Low expression|	positive|TC 5%, IC<1%	1,9	MSS
361	71	Male	KRAS:c.35G>A (p.Gly12Asp)|FANCL:c.1110_1113dup (p.Thr372AsnfsTer13)|CDKN2A:c.181G>T (p.Glu61Ter)|SMARCA4:c.2741delT (p.Leu914ArgfsTer36)|TP53:c.527G>A (p.Cys176Tyr)|Microsatellite Instability (MSI):MSS|Loss of Heterozygosity (LOH):LOHH 16,2%|PD-L1 expression:positive TC 1%, IC<1%|Tumor Mutational Burden (TMB):6,64 Muts/MB|ERCC1:Low expression|TYMS:Low expression|	positive|TC 1%, IC<1%	6,64	MSS

362	75	Female	KRAS:c.35G>T (p.Gly12Val)|TP53:c.817C>T (p.Arg273Cys)|SMAD4:c.1333 C>T (p.Arg445Ter)|RB1:c.219_220 dup (p.Ala74GlufsTer4)|Microsatellite Instability (MSI):MSS|PD-L1 expression:negative TC<1%, IC<1%|Tumor Mutational Burden (TMB):1,9 Muts/MB|	negative| TC<1%, IC<1%	1,9	MSS
363	39	Male	KRAS:c.34G>C (p.Gly12Arg)|TP53:c.586C>T (p.Arg196Ter)|Microsatellite Instability (MSI):MSS|PD-L1 expression:negative TC<1%, IC<1%|Tumor Mutational Burden (TMB):0 Muts/MB|	negative| TC<1%, IC<1%	0	MSS
364	51	Female	KRAS:c.35G>A (p.Gly12Asp)|TP53:c.673-1G>A (splicing)|KMT2C:c.3869delG (p.Arg1290LysfsTer13)|Microsatellite Instability (MSI):MSS|PD-L1 expression:negative TC<1%, IC<1%|Tumor Mutational Burden (TMB):1,9 Muts/MB|ERCC1:Low expression|TYMS:Low expression|	negative| TC<1%, IC<1%	1,9	MSS

365	81	Female	KRAS:c.35G>A (p.Gly12Asp)|TP53:c.524G>A (p.Arg175His)|Microsatellite Instability (MSI):MSS|PD-L1 expression:positive TC<1% IC 5%|Tumor Mutational Burden (TMB):1.9 Muts/MB|	positive|TC<1% IC 5%	1,9	MSS
366	41	Female	KRAS:c.182_184delinsGAA (p.Gln61_Glu62delinsArgLys)|TP53:c.319_320dup (p.Gly108ThrfsTer16)|Microsatellite Instability (MSI):MSS|Tumor Mutational Burden (TMB):1.9 Muts/MB|		1,9	MSS
367	58	Male	KRAS:c.35G>A (p.Gly12Asp)|Microsatellite Instability (MSI):MSS|PD-L1 expression:negative TC<1%, IC<1%|Tumor Mutational Burden (TMB):6,67 Muts/MB|	negative|TC<1%, IC<1%	6,67	MSS
368	41	Female	KRAS:c.34G>C (p.Gly12Arg)|TP53:c.817C>T (p.Arg273Cys)|ARID1A:c.3664_3668delGGGCGinsAGC (p.Gly1222SerfsTer5)|Microsatellite Instability (MSI):MSS|PD-L1 expression:negative TC<1%, IC<1%|Tumor Mutational Burden (TMB):2,84 Muts/MB|	negative|TC<1%, IC<1%	2,84	MSS

369	48	Male	KRAS:c.34G>C (p.Gly12Arg)|TP53:c.916C>T (p.Arg306Ter)|Microsatellite Instability (MSI):MSS|PD-L1 expression:negative TC<1% IC<1%|Tumor Mutational Burden (TMB):1.91 Muts/MB|	negative| TC<1% IC<1%	1,91	MSS
370	71	Female	KRAS:c.35G>A (p.Gly12Asp)|TP53:c.725G>T (p.Cys242Phe)|Microsatellite Instability (MSI):MSS|PD-L1 expression:negative TC<1%, IC<1%|Tumor Mutational Burden (TMB):4,76 Muts/MB|	negative| TC<1%, IC<1%	4,76	MSS
371	71	Female	KRAS:c.34G>C (p.Gly12Arg)|SMAD4:c.1052A>T (p.Asp351Val)|RNF43:c.433C>T (p.Arg145Ter)|TP53:c.637C>T (p.Arg213Ter)|AURKA:c.1187_1188del (p.Lys396ArgfsTer4)|Microsatellite Instability (MSI):MSS|PD-L1 expression:negative TC<1% IC<1%|Tumor Mutational Burden (TMB):6.65 Muts/MB|	negative| TC<1% IC<1%	6,65	MSS

372	74	Male	KRAS:c.35G>T (p.Gly12Val)|CDKN2A:c.153_154insG (p.Met52AspfsTer68)|ARID1A:c.5202T>A (p.Tyr1734Ter)|TP53:c.743G>A (p.Arg248Gln)|RASA1:c.1288C>T (p.Gln430Ter)|Microsatellite Instability (MSI):MSS|PD-L1 expression:positive TC<1% IC 10%|Tumor Mutational Burden (TMB):7.62 Muts/MB|	positive|TC<1% IC 10%	7,62	MSS
373	58	Female	Microsatellite Instability (MSI):MSS|PD-L1 expression:negative TC<1%, IC<1%|Tumor Mutational Burden (TMB): Muts/MB|	negative| TC<1%, IC<1%		MSS
374	67	Male	KRAS:c.34G>C (p.Gly12Arg)|CDKN2A:c.45G>A (p.Trp15Ter)|KDM6A:c.2588_2589insA (p.Ser864GlufsTer11)|TP53:c.713G>A (p.Cys238Tyr)|Microsatellite Instability (MSI):MSS|PD-L1 expression:negative TC<1% IC<1% |Tumor Mutational Burden (TMB):2.85 Muts/MB|	negative| TC<1% IC<1%	2,85	MSS

<u>375</u>	60	Male	KRAS:c.35G>T (p.Gly12Val)|PIK3CA:c.3139C>T (p.His1047Tyr)|MAP2K7:c.289C>T (p.Gln97Ter)|TP53:c.734G>A (p.Gly245Asp)|Microsatellite Instability (MSI):MSS|Loss of Heterozygosity (LOH):LOHL 12.67 %|PD-L1 expression:negative TC<1% IC<1%|Tumor Mutational Burden (TMB):2.85 Muts/MB|	negative| TC<1% IC<1%	2,85	MSS
<u>376</u>	59	Male	KRAS:c.35G>T (p.Gly12Val)|CDKN2A:c.174_207del (p.Val59ProfsTer76)|TP53:c.723delC (p.Cys242AlafsTer5)|CCND2:amplification|Microsatellite Instability (MSI):MSS|PD-L1 expression:negative TC<1% IC<1%|Tumor Mutational Burden (TMB):2.87 Muts/MB|ERCC1:Low expression|TYMS:Low expression|UGT1A1:HETEROZYGOUS (6/7)|DPD:WT|	negative| TC<1% IC<1%	2,87	MSS

377	37	Female	KRAS:c.35G>T (p.Gly12Val)|SMAD4:c.431C>A (p.Ser144Ter)|TP53:c.469G>C (p.Val157Leu)|RNF43:c.1624_1651del (p.Gln543AlafsTer148)|Microsatellite Instability (MSI):MSS|PD-L1 expression:negative TC<1%, IC<1%|Tumor Mutational Burden (TMB):4,76 Muts/MB|	negative|TC<1%, IC<1%	4,76	MSS
378	61	Male	KRAS:c.38G>A (p.Gly13Asp)|BAP1:c.783+2T>C|CDKN2A:c.151-1G>T (splicing)|Microsatellite Instability (MSI):MSS|PD-L1 expression:positive TC<1%, IC 2%|Tumor Mutational Burden (TMB):5,71 Muts/MB|	positive|TC<1%, IC 2%	5,71	MSS
379	45	Male	KRAS:c.182_183delinsTG (p.Gln61Leu)|Microsatellite Instability (MSI):MSS|PD-L1 expression:negative TC<1% IC<1%|Tumor Mutational Burden (TMB):1.93 Muts/MB|	negative|TC<1% IC<1%	1,93	MSS

380	55	Male	BRCA1:gene duplication|KRAS:c.35G>A (p.Gly12Asp)|CDKN2A:c.329G>A (p.Trp110Ter)|TP53:c.637C>T (p.Arg213Ter)|Microsatellite Instability (MSI):MSS|Loss of Heterozygosity (LOH):LOHH 24.82 %|PD-L1 expression:negative TC<1% IC<1%|Tumor Mutational Burden (TMB):3.82 Muts/MB|	negative| TC<1% IC<1%	3,82	MSS
381	36	Male	KRAS:c.35G>T (p.Gly12Val)|SMAD4:c.170_173del (p.Leu57Ter)|TP53:c.646G>T (p.Val216Leu)|Microsatellite Instability (MSI):MSS|Tumor Mutational Burden (TMB):1,9 Muts/MB|		1,9	MSS
382	49	Male	PALB2:c.2257C>T (p.Arg753Ter)|KRAS:c.35G>A (p.Gly12Asp)|Microsatellite Instability (MSI):MSS|PD-L1 expression:negative TC<1%, IC<1%|Tumor Mutational Burden (TMB):3,8 Muts/MB|	negative| TC<1%, IC<1%	3,8	MSS
383	66	Female	KRAS:c.34G>C (p.Gly12Arg)|TP53:c.820G>T (p.Val274Phe)|Microsatellite Instability (MSI):MSS|PD-L1 expression:positive TC<1%, IC<1%|Tumor Mutational Burden (TMB):4,75 Muts/MB|	positive|TC<1%, IC<1%	4,75	MSS

384	59	Female	WT1:c.662-1G>T (splicing)|TP53:c.673-2A>G (splicing)|Microsatellite Instability (MSI):MSS|Loss of Heterozygosity (LOH):LOHH 61.85 %|PD-L1 expression:positive TC1% IC<1%|Tumor Mutational Burden (TMB):5.71 Muts/MB|	positive|TC1% IC<1%	5,71	MSS
385	63	Female	KRAS:c.35G>A (p.Gly12Asp)|Microsatellite Instability (MSI):MSS|Tumor Mutational Burden (TMB):7.64 Muts/MB|		7,64	MSS
386	84	Female	KRAS:c.34G>C (p.Gly12Arg)|TP53:c.376-2A>G (splicing)|Microsatellite Instability (MSI):MSS|PD-L1 expression:negative TC<1%, IC<1%|Tumor Mutational Burden (TMB):3,8 Muts/MB|	negative| TC<1%, IC<1%	3,8	MSS
387	80	Male	KRAS:c.34G>C (p.Gly12Arg)|			
388	86	Female	KRAS:c.35G>T (p.Gly12Val)|PIK3CA:c.1636C>G (p.Gln546Glu)|ARID2:c.1750G>T (p.Glu584Ter)|TP53:c.844C>T (p.Arg282Trp)|Microsatellite Instability (MSI):MSS|PD-L1 expression:negative TC<1%, IC<1%|Tumor Mutational Burden (TMB):3,82 Muts/MB|	negative| TC<1%, IC<1%	3,82	MSS

389	59	Male	KRAS:c.35G>T (p.Gly12Val)|ARID1A:c.6105_6106dup (p.Glu2036GlyfsTer7)|SMAD4:c.1037delC (p.Pro346LeufsTer38)|TP53:c.596G>T (p.Gly199Val)|Microsatellite Instability (MSI):MSS|Tumor Mutational Burden (TMB):3.8 Muts/MB|		3,8	MSS
390	73	Male	KRAS:c.35G>A (p.Gly12Asp)|CDKN2A:c.130delT (p.Tyr44ThrfsTer9)|TP53:c.711G>T (p.Met237Ile)|Microsatellite Instability (MSI):MSS|PD-L1 expression:positive TC 30% IC<1%|Tumor Mutational Burden (TMB):FAIL Muts/MB|	positive|TC 30% IC<1%	FAIL	MSS
391	48	Male	FANCL:c.2T>C (p.Ala2_Met74del)|KRAS:c.35G>A (p.Gly12Asp)|TP53:c.660T>G (p.Tyr220Ter)|Microsatellite Instability (MSI):MSS|PD-L1 expression:negative TC<1% IC<1% |Tumor Mutational Burden (TMB):7.6 Muts/MB|	negative| TC<1% IC<1%	7,6	MSS

392	62	Male	ATM:c.9170G>C (p.Ter3057Ser)|GNAS:c.601C>T (p.Arg201Cys)|Microsatellite Instability (MSI):MSS|PD-L1 expression:negative TC<1% IC<1% |Tumor Mutational Burden (TMB):3.9 Muts/MB|ERCC1:Low expression|TYMS:Low expression|	negative| TC<1% IC<1%	3,9	MSS
393	82	Male	KRAS:c.35G>A (p.Gly12Asp)|AKT1:c.49G>A (p.Glu17Lys)|CTNNB1:c.97T>G (p.Ser33Ala)|Microsatellite Instability (MSI):MSS|PD-L1 expression:negative TC<1% IC<1% |Tumor Mutational Burden (TMB):12.34 Muts/MB|	negative| TC<1% IC<1%	12,34	MSS
394	65	Male	Microsatellite Instability (MSI):MSS|Tumor Mutational Burden (TMB):1,92 Muts/MB|ERCC1:High expression|TYMS:Low expression|UGT1A1:WT (6/6)|DPD:WT|		1,92	MSS

395	79	Female	KRAS:c.182A>G (p.Gln61Arg)|TP53:c.586C>T (p.Arg196Ter)|RNF43:c.1111C>T (p.Arg371Ter)|PPP2R1A:c.548G>A (p.Arg183Gln)|FGFR3 fusion:FGFR3(17) - TACC3(14)|Microsatellite Instability (MSI):MSS|PD-L1 expression:positive TC<1%, IC 5%|Tumor Mutational Burden (TMB):6,63 Muts/MB|	positive|TC<1%, IC 5%	6,63	MSS
396	61	Female	KRAS c.35G>A p.Gly12Asp |SF3B1 c.2098A>G p.Lys700Glu| TP53 c.451C>T p.Pro151Ser1	positive|TC10% IC<1%	0	MSS
397	69	Male	KRAS:c.34G>C (p.Gly12Arg)|KRAS:c.183A>C (p.Gln61His)|PTEN:c.987_990delTAAA (p.Asn329LysfsTer14)|PTEN:c.955_958delACTT (p.Thr319Ter)|TP53:c.380C>T (p.Ser127Phe)|TP53:c.701A>G (p.Tyr234Cys)|	negative| TC<1% IC<1%	16,38	MSS
398	65	Female	KRAS:c.35G>A (p.Gly12Asp)|ERBB2:amplification	negative| TC<1%, IC<1%	0	MSS
399	77	Female	KRAS:c.35G>A (p.Gly12Asp)|ARID2:c.1189C>T (p.Gln397Ter)|TP53:c.683_686del (p.Asp228ValfsTer18)|	negative| TC<1% IC<1%	4,78	MSS

<table>
<tr><td>400</td><td>65</td><td>Male</td><td>KRAS:c.35G>A
(p.Gly12Asp)|TP53:c.743G>A
(p.Arg248Gln)</td><td>negative|
TC<1%
IC<1%</td><td>5,72</td><td>MSS</td></tr>
</table>
