# Supplementary figures and images for: Digging into the NGS Information from a Large-Scale South European Population with Metastatic/Unresectable Pancreatic Ductal Adenocarcinoma: A Real-World Genomic Depiction
Source: Cancers (Basel). 2023 Dec 19;16(1):2. doi: 10.3390/cancers16010002 (PMC10778112; doi:10.3390/cancers16010002)

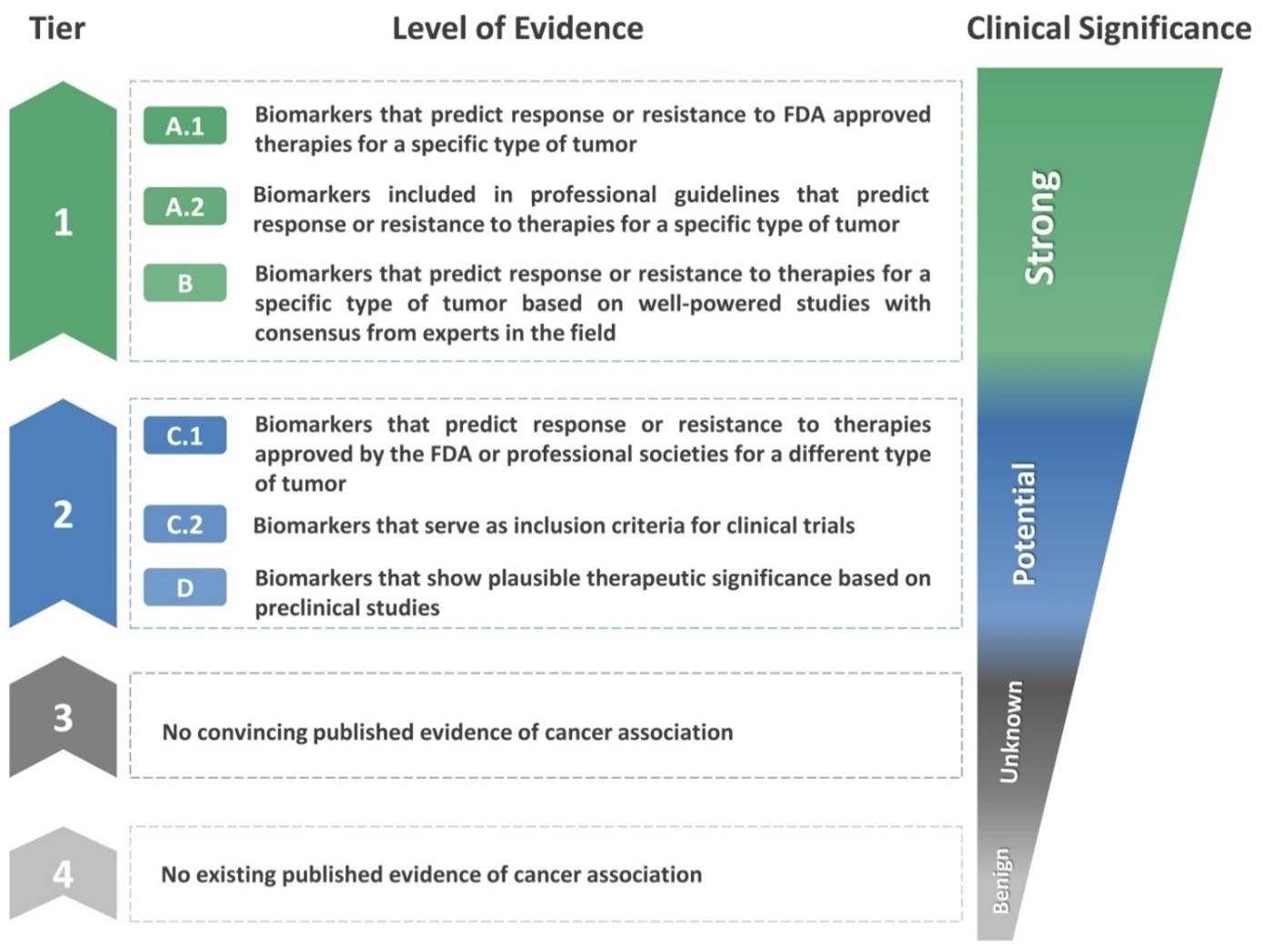

Supplement: Supplementary file 1 [file cancers-16-00002-s001.zip › Figure S1.jpg]
